# Highly enantioselective reduction of benzophenones by engineered *Geotrichum candidum* alcohol dehydrogenase

**DOI:** 10.1007/s00253-026-13717-0

**Published:** 2026-01-29

**Authors:** Zhongyao Tang, Guillermo Germán Otárola Tejada, Afifa Ayu Koesoema, Tomoko Matsuda

**Affiliations:** 1 Department of Life Science and Technology, School of Life Science and Technology, Institute of Science Tokyo, 4259 Nagatsuta-Cho Midori-Ku, Yokohama, 226-8501 Japan; 2 Department of Organic and Inorganic Chemistry, University of Alcalá, Ctra. Madrid-Barcelona Km 33100, Alcalá de Henares, 28805 Madrid, Spain

**Keywords:** Biocatalyst, Medium-chain dehydrogenase/reductase, Enzyme engineering, Asymmetric reduction, Benzophenones, Chiral diaryl methanols

## Abstract

**Abstract:**

Biocatalytic approaches have gained increasing attention as sustainable alternatives to metal-catalyzed asymmetric reductions of ketones to obtain enantiopure alcohols, important intermediates for pharmaceutical synthesis. For example, enzyme-catalyzed reduction of substituted benzophenone analogs to produce chiral diaryl methanols has attracted interest, as they are the key intermediates in the synthesis of antihistamines. However, benzophenone analogs are difficult to be reduced by enzymes due to steric hindrance. Moreover, the similarities between the two groups adjacent to the carbonyl group make achieving high enantioselectivity in reduction challenging. In this study, we examined the reduction of benzophenone and its analogs by *Geotrichum candidum* acetophenone reductase (*Gc*APRD). However, the wild type did not exhibit activity toward benzophenone due to the substrate’s bulkiness. Then, two mutants of *Gc*APRD (Trp288Ala and Phe56Ile/Trp288Ala) were applied to catalyze the reduction of benzophenone, resulting in high reduction yield (≥ 80%). In addition, both mutants exhibited catalytic activity toward methyl- and halogen-substituted benzophenones, especially toward 3- and 4-substituted substrates. Regarding enantioselectivity, Trp288Ala generally reduced both 3- and 4-substituted substrates to (*R*)-alcohols with up to 97% *ee*. In contrast, Phe56Ile/Trp288Ala reduced 3-substituted substrates to (*R*)-alcohols with up to 89% *ee* but reduced 4-substituted substrates to (*S*)-alcohols with up to 92% *ee*. At last, the reduction mechanism was investigated using molecular docking simulations.

**Key points:**

• *GcAPRD mutants exhibited catalytic performance toward benzophenone analogs.*

• *GcAPRD Phe56Ile/Trp288Ala exhibited substituent-dependent enantioselectivity.*

• *Introducing Phe56Ile into GcAPRD Trp288Ala resulted in a clear enantiopreference.*

**Graphical Abstract:**

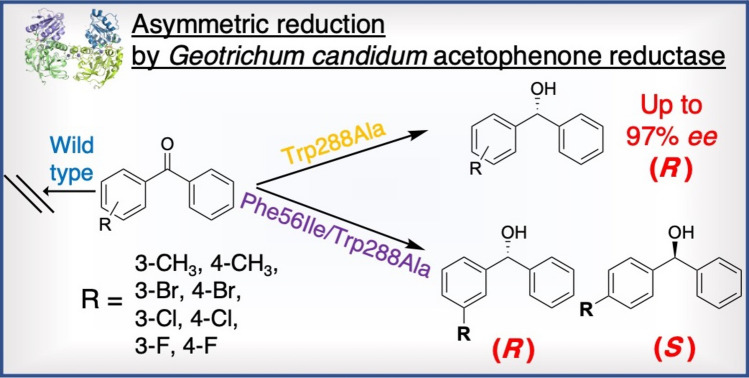

**Supplementary Information:**

The online version contains supplementary material available at 10.1007/s00253-026-13717-0.

## Introduction

Chiral alcohols are important building blocks for the synthesis of various pharmaceutical molecules. Presently, the major synthetic method for these chiral compounds relies on the asymmetric reduction of the corresponding ketones, catalyzed by precise metal catalysts (e.g., Cu, Ir, Ru, Rh) typically under a hydrogen atmosphere or through transfer hydrogenation in organic solvents (Fujii et al. 1996; Chen et al. 2003; Elford et al. 2011; Tao et al. 2012; Gaykar et al. 2018; Zhang et al. 2019; Guan et al. 2025; Zheng et al. 2025). However, this approach has several drawbacks, including potential environmental and safety risks. For these reasons, using biocatalysts instead of chemical catalysts has attracted increasing attention in recent years (Bornscheuer et al. 2012; Alemán and Cabrera 2013; Nealon et al. 2015; Santi et al. 2021; Winkler et al. 2021; Sardauna et al. 2023; Reisenbauer et al. 2024). Moreover, high catalytic activity and, in particular, excellent enantioselectivity have been demonstrated mainly using hydrolases followed by oxidoreductases (Musa et al. 2018; Sheldon and Woodley 2018; Tanaka et al. 2020; Zhou et al. 2020; Wu et al. 2021b; Tang et al. 2024a; Farhan et al. 2025).

Among various oxidoreductases, a medium-chain dehydrogenase/reductase (MDR) from *Geotrichum candidum* NBRC 4597 (*G. candidum* acetophenone reductase, *Gc*APRD; PDB ID: 6ISV) has exhibited excellent activity and enantioselectivity in the asymmetric reduction of aliphatic and aromatic ketones following Prelog’s rule (Prelog 1964; Nakata et al. 2010; Yamamoto et al. 2013; Koesoema et al. 2019b, 2019a, 2020b, 2020a). Moreover, three *Gc*APRD mutants, Trp288Ala, Phe56Ile/Trp288Ala, and Phe56Ala/Trp288Ala, have been shown to efficiently catalyze the asymmetric reduction of 2-benzoylpyridine analogs and to produce the corresponding (*R*)-alcohols, which are key intermediates in the synthesis of antihistamine drugs (Tang et al. 2024b). This subject has rarely been challenged using other MDRs, and the superiority of the *Gc*APRD mutants over other MDRs for the reduction of 2-benzoylpyridines has been clearly demonstrated.

In this study, we focused on the *Gc*APRD-catalyzed reduction of benzophenone analogs with similar structures to 2-benzoylpyridine analogs. The products of these reductions are also intermediates in the synthesis of antihistamines, such as diphenhydramine, orphenadrine, and cloperastine (Thompson et al. 2019; Wu et al. 2021a; Kammoun et al. 2023). However, steric hindrance and low solubility reduce the reactivity of benzophenones, making enzymatic reductions much more challenging than those of 2-benzoylpyridines (Sun et al. 2016). Moreover, the similarity between the phenyl and substituted-phenyl groups and the long distance of the substituent from the carbonyl group make it difficult to achieve high enantioselectivity.

To the best of our knowledge, only a few alcohol dehydrogenases (ADHs) from the short-chain dehydrogenase/reductase (SDR) family, such as ADH from *Kluyveromyces polyspora* (*Kp*ADH), ADH from *Lactobacillus kefiri* (*Lk*ADH), and ketoreductase from *Kluyveromyces marxianus* CBS4857 (*Km*CR2), have been reported to reduce benzophenones (Wang et al. 2018; Xu et al. 2018, 2020; Zhou et al. 2018; Li et al. 2019; Wu et al. 2020, 2021a). In contrast, within the MDR family, only ADH from *Thermoanaerobacter brockii* (*Tb*SADH) has been studied for the reduction of only two substrates, 4-chlorobenzophenone and 4-nitrobenzophenone (Liu et al. 2019; Qu et al. 2019). Therefore, further investigations of robust MDRs are needed to increase the diversity of enzymes capable of catalyzing the reduction of benzophenones.

**Fig. 1:**
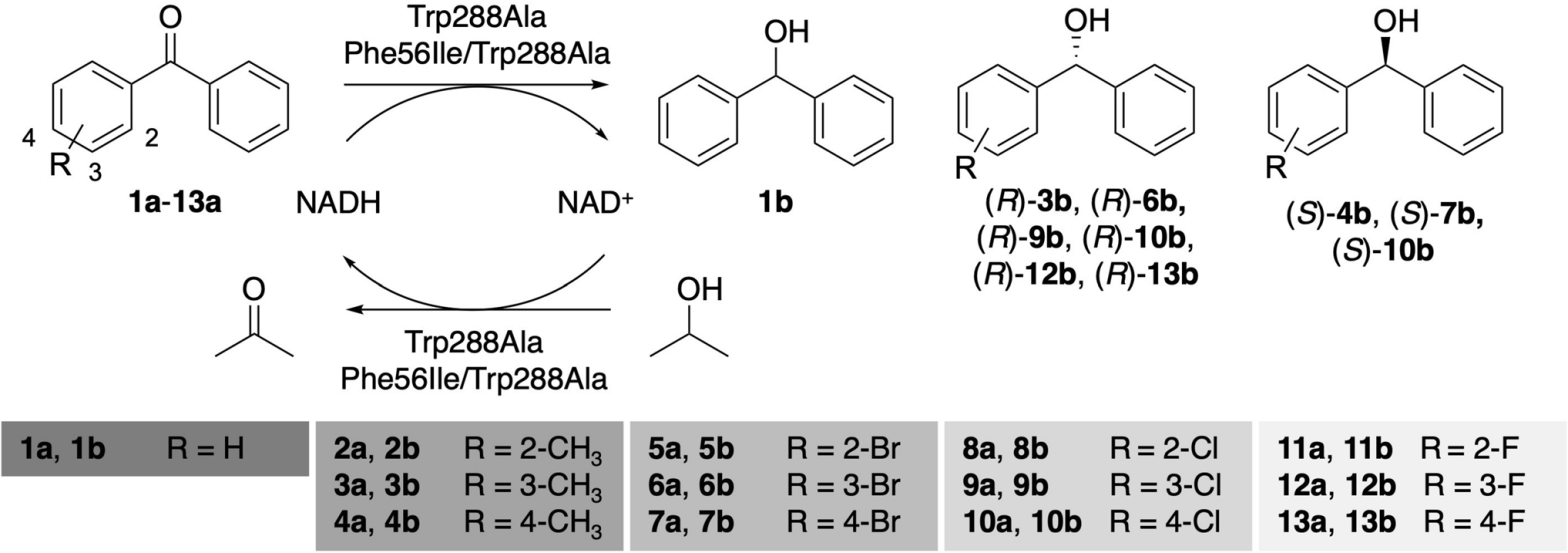
Reduction of benzophenone analogs by *Gc*APRD mutants

To address this issue, we investigated the reduction of benzophenone analogs (**1a**–**13a**) using *Gc*APRD from the MDR family as a catalyst (Fig. 1). Although the wild type did not exhibit activity toward benzophenone, two *Gc*APRD mutants, Trp288Ala and Phe56Ile/Trp288Ala, were found to be able to reduce benzophenones enantioselectively. Subsequently, we conducted a molecular docking simulation to explain the observed activity, substrate specificity, and enantioselectivity.

## Materials and methods

### Chemicals

Most chemicals, including ketones (**1a**–**13a**) and alcohol standards (**1b**, *rac*-**4b**, *rac*-**7b**, *rac*-**9b**, and *rac*-**10b**), were purchased from Nacalai Tesque (Kyoto, Japan), Wako (Osaka, Japan), Tokyo Chemical Industry (Tokyo, Japan), Bio-Rad (Hercules, CA, USA), and Sigma-Aldrich (St. Louis, MO, USA). Other racemic alcohol standards (*rac*-**3b**, *rac*-**6b**, *rac*-**12b**, and *rac*-**13b**) were synthesized by sodium borohydride reduction of the corresponding ketones. Detailed information is provided in the supplementary information (Sect. 1).

### Biocatalyst preparation

*E. coli* whole cells expressing *Gc*APRD wild type and mutants were prepared as previously reported procedures by using *E. coli* Rosetta™(DE3)plysS (Invitrogen, Carlsbad, CA, USA) with pET-21b(+)-*Gc*APRD wild type or mutants (Yamamoto et al. 2013; Koesoema et al. 2019a; Tang et al. 2024b). After induction of protein overexpression with isopropyl β-d-1-thiogalactopyranoside (IPTG, 1.0 mM), the cells were harvested by centrifugation and used for subsequent reactions.

### Reduction of benzophenone analogs by *Gc*APRD wild type, Trp288Ala, and Phe56Ile/Trp288Ala

**Fig. 2:**
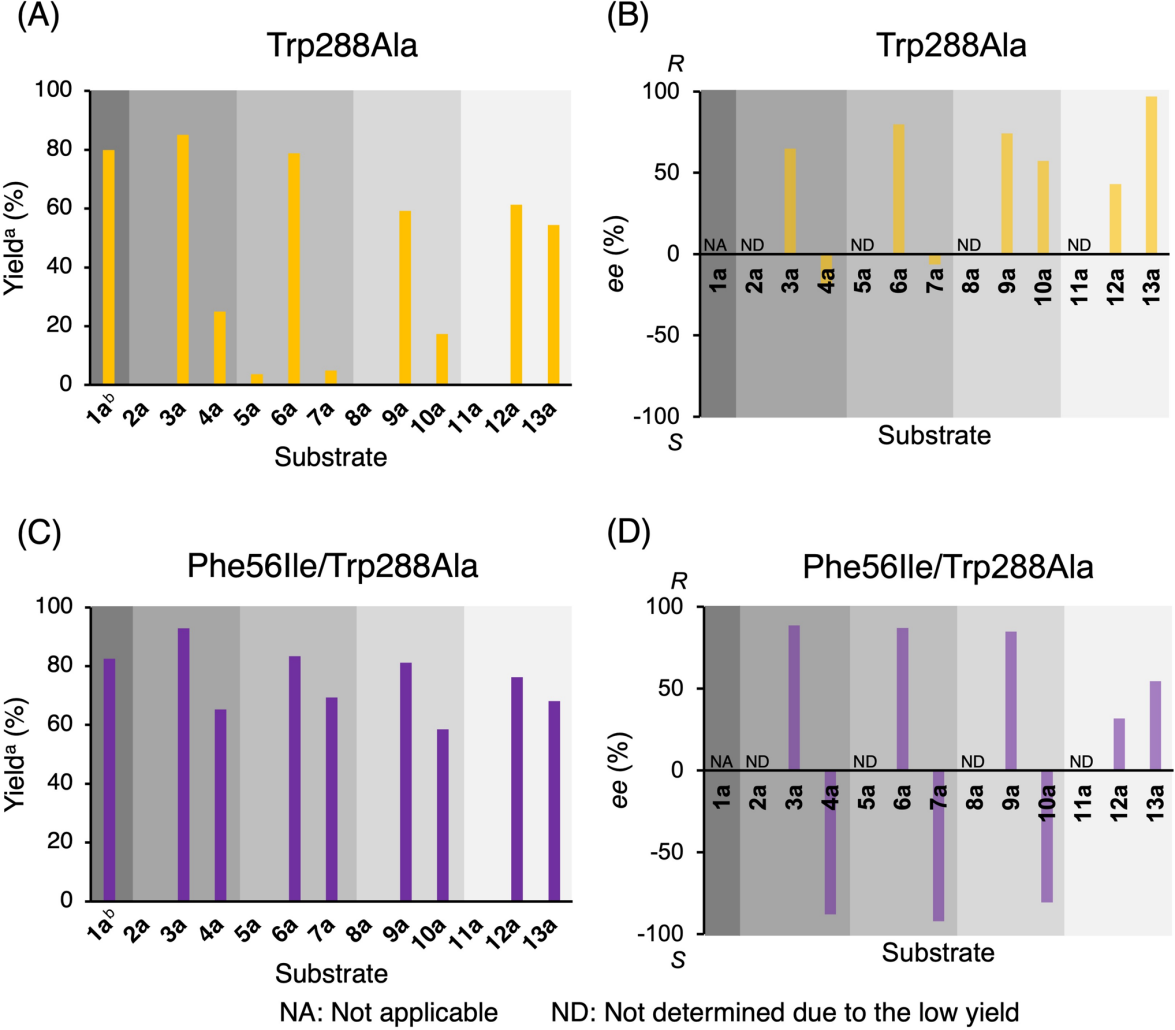
Reductions of **1a**-**13a** by *Gc*APRD mutants: (**A**) yield and (**B**) enantioselectivity for the reaction by Trp288Ala, (**C**) yield and (**D**) enantioselectivity for the reaction by Phe56Ile/Trp288Ala. Small-scale reductions were performed in HEPES-NaOH buffer (100 mM, pH 7.2, 12.0 mL) consisting of 2-propanol (15% v/v), substrate (2.5 mM), and whole cells (2.0 g wet weight) at 30 ℃ with a shaking speed of 250 rpm for 24 h. ^a^The yield was determined by H-NMR analysis. The signal of the product used for the calculation was at 5.8 ppm (1H, singlet). The signal of the internal standard, 1,4-dioxane, used for the calculation was at 3.7 ppm (8H, singlet). The isolated yields shown in the supplementary information (Sect. 2) matched the H-NMR yields. ^b^The yields of **1a** were taken from Table 1

Small-scale reductions of **1a**–**13a** were performed in HEPES–NaOH buffer (100 mM, pH 7.2, 12.0 mL) consisting of 2-propanol (15% v/v), substrate (2.5 mM), and whole cells (2.0 g wet weight) at 30 ℃ with a shaking speed of 250 rpm for 24 h. The products were extracted with diethyl ether three times and concentrated under reduced pressure. Product yields were determined by ^1^H-NMR analysis (400 MHz Bruker Biospin Avance III 400 A, Bruker, USA) using the crude extract. Silica gel column chromatography (hexane/ethyl acetate, 5:1) was performed to obtain the corresponding products. The products were characterized by ^1^H-NMR analysis, and enantiomeric excess (*ee*) was determined by chiral HPLC analysis (LC-20AD with SPD-20A UV–vis detector, Shimadzu, Japan), equipped with a chiral column. The ^1^H-NMR spectra agreed with those reported in the literature. The HPLC analysis conditions and the retention times of the *R* and *S* enantiomers are listed in Table S1. ^1^H-NMR yield and *ee* values are provided in Table 1, Fig. 2, and Table S2. Detailed information, including isolated yields, is provided in the supplementary information (Sect. 2)*.*

Scaled-up reductions of **3a** and **9a** by *Gc*APRD Phe56Ile/Trp288Ala were also performed in HEPES–NaOH buffer (100 mM, pH 7.2, 120 mL) consisting of 2-propanol (15% v/v), **3a** (65.9 mg, 2.8 mM) or **9a** (74.7 mg, 2.9 mM), and whole cells (6.0 g wet weight) with the above procedure. Detailed information is provided in the supplementary information (Sect. 3)*.*

### Docking simulation procedure

Docking simulations were performed for the tested substrates as ligands with the *Gc*APRD mutants (Trp288Ala and Phe56Ile/Trp288Ala) NAD^+^ complex model as receptors. Mutations were introduced in the crystal structure of *Gc*APRD wild type complexed with NAD^+^ (PDB ID: 6ISV) using PyMOL Molecular Graphics System version 2.5.2 (Schrödinger, LLC). The SDF files for ligands were obtained from the PubChem database. Receptors and ligands were prepared using Schrödinger Maestro version 12.9. The volume of the cubic grid box centered on the receptor chain A catalytic zinc was set to 10,648 Å^3^. The Glide docking was performed in standard precision (SP) mode with default parameters, utilizing the OPLS4 force field, which allows for ligand flexibility. The NAD^+^ cofactor and Zn^2+^ were retained throughout all simulations. Ligand strain energies were calculated using default settings, with implicit water solvation applied to approximate physiological conditions.

The induced fit docking (IFD) was in extra precision (XP) mode with default parameters and the OPLS4 force field, using the same grid box settings as in the Glide docking. Side chains within 5 Å of the ligand were allowed to move to accommodate the binding pose. Strain energies of IFD results were not calculated due to methodological incompatibility.

The productive poses were determined according to previously reported distance and angle restraint parameters (Koesoema et al. 2019b). Detailed descriptions are provided in the supplementary information (Fig. S1)*.*

## Results

### Asymmetric reductions of benzophenone analogs by *Gc*APRD wild type, Trp288Ala, and Phe56Ile/Trp288Ala

To evaluate the catalytic performance of *Gc*APRD wild type and mutants, Trp288Ala and Phe56Ile/Trp288Ala, **1a** was used as a representative substrate in whole-cell-catalyzed reductions. 2-Propanol (15% v/v) was added to recycle the cofactor and to solubilize the substrate. This enzyme can tolerate up to 15% v/v 2-propanol, allowing the highly hydrophobic substrate to be used successfully. The yields of reduction are listed in Table 1. After a reaction time of 24 h, no reaction occurred with the wild type (yield < 0.1%). In contrast, under the same reaction conditions for the same reaction time, Trp288Ala and Phe56Ile/Trp288Ala afforded significantly higher yields of 80% and 83%, respectively. This result demonstrated the potential of Trp288Ala and Phe56Ile/Trp288Ala to catalyze the reduction of other benzophenone analogs.

**Table 1:** The reduction of **1a** by *Gc*APRD wild type and mutants

Enzyme	Wild type	Trp288Ala	Phe56Ile/Trp288Ala
Yield^a^ (%)	< 0.1	80	83

Small-scale reductions were performed in a HEPES–NaOH buffer (100 mM, pH 7.2, 12.0 mL) consisting of 2-propanol (15% v/v), substrate (2.5 mM), and whole cells (2.0 g wet weight) at 30 ℃, with a shaking speed of 250 rpm for 24 h

^a^The yield was determined by ^1^H−NMR analysis. The signal of the product used for the calculation was at 5.8 ppm (1H, singlet). The signal of the internal standard, 1,4−dioxane, used for the calculation was at 3.7 ppm (8H, singlet)

Next, methyl- (**2a**–**4a**), bromo- (**5a**–**7a**), chloro- (**8a**–**10a**), and fluoro- (**11a**–**13a**) substituted benzophenones were used as substrates to investigate the substrate specificity and enantioselectivity of Trp288Ala and Phe56Ile/Trp288Ala under the same reaction conditions for 24 h (Fig. 2 and Table S2). Both mutants exhibited catalytic activity toward these benzophenones depending on the position and kind of substituents (Figs. 2A and 2C). Compared with Trp288Ala, Phe56Ile/Trp288Ala generally showed higher yields. However, neither mutant catalyzed the reduction of 2-substituted substrates (**2a**, **5a**, **8a**, and **11a**) efficiently. When 3- and 4-substituted substrates were compared, both mutants showed higher yields for the reactions with 3-substituted substrates than with 4-substituted substrates (for both mutants, 3-CH_3_ > 4-CH_3_, 3-Br > 4-Br, 3-Cl > 4-Cl, 3-F > 4-F). The highest yields were obtained using **3a** (3-CH_3_), reaching 85% for Trp288Ala and 93% for Phe56Ile/Trp288Ala. Regarding various 3-substituted substrates, for both mutants, the yields generally increased as the hydrophobicity of the substituent on the 3-position increased (3-CH_3_ > 3-Br > 3-Cl ≈ 3-F). Meanwhile, regarding various 4-substituted substrates, the yields of Trp288Ala-catalyzed reactions were influenced by the substituent (4-F > 4-CH_3_ > 4-Cl > 4-Br), but the substituent did not significantly affect the yields of Phe56Ile/Trp288Ala-catalyzed reactions.

Regarding enantioselectivity, Trp288Ala generally produced (*R*)-alcohols following anti-Prelog’s rule (Prelog 1964) (Fig. 2B). Particularly, Trp288Ala produced (*R*)-**13b** (4-F) with 97% *ee*, which was the highest enantioselectivity among all the reactions tested. However, for other 4-substituted substrates, the enantioselectivity of Trp288Ala was generally lower than that of the corresponding 3-substituted substrates.

In contrast, Phe56Ile/Trp288Ala showed (*R*)-enantioselectivity toward 3-substituted substrates but showed (*S*)-enantioselectivity toward 4-substituted substrates, except for **13a** (Fig. 2D). For example, (*R*)-**3b** with 89% *ee* was produced, while (*S*)-**4b** with 88% *ee* was produced. Similarly, (*R*)-**6b** with 87% *ee*, (*S*)-**7b** with 92% *ee*, (*R*)-**9b** with 85% *ee*, and (*S*)-**10b** with 81% *ee* were obtained. Notably, Phe56Ile/Trp288Ala exhibited higher enantioselectivity than Trp288Ala for most tested substrates, except for **12a** and **13a**.

### Scaled-up reductions of 3a and 9a by *Gc*APRD Phe56Ile/Trp288Ala

The isolation of the product in the small scale was performed for almost all products. To further explore the synthetic applicability of Phe56Ile/Trp288Ala, **3a** was reduced on a larger scale, since among the tested substrates it gave the highest yield in the small-scale reactions. The reaction resulted in 96% isolated yield (63.6 mg), and 92% *ee* (*R*), both slightly higher than those observed on the small scale.

In addition, to provide a comparison between 3-methyl (**3a**) and 3-chloro (**9a**) substitution, **9a** was selected as a representative substrate. The reaction resulted in 83% isolated yield (62.4 mg), and 83% *ee* (*R*), consistent with the high yield and enantioselectivity observed on the small scale. These results demonstrated that Phe56Ile/Trp288Ala maintained high catalytic performance in the scaled-up reaction.

## Docking simulation of *Gc*APRD

Docking simulations were performed using **1a**, 3-substituted (**3a**, **6a**, **9a**, and **12a**) and 4-substituted (**4a**, **7a**, **10a**, and **13a**) substrates as ligands and *Gc*APRD wild type, Trp288Ala and Phe56Ile/Trp288Ala NAD^+^ complex as receptors. Under the same docking simulation conditions, no binding poses of **1a** were obtained in *Gc*APRD wild type, as the ligand could not enter the binding site. On the other hand, the productive binding poses of **1a** and the pro-*S* and pro-*R* binding poses for 3-substituted ligands (**3a**, **6a**, **9a**, and **12a**) and 4-substituted ligands (**4a**, **7a**, **10a**, and **13a**) were obtained in both *Gc*APRD mutants, mostly satisfying the restraint parameters defined for catalysis, although the hydride attack angles slightly deviated from the ideal range (Tables S3 and S4). In the pro-*S* pose, the substituted phenyl ring was generally oriented toward the pocket entrance, whereas in the pro-*R* pose, it was located toward the engineered small pocket (Figs. S3–S6).

In Trp288Ala, for both pro-*S* and pro-*R* binding poses, the substituted phenyl or phenyl ring of the ligand was located between Phe56 and Phe287, respectively, causing spatial constraints (Figs. S3 and S4). As a result, the strain energies of ligands in Trp288Ala were generally increased, typically ranging from 2.4 to 5.2 kcal/mol, except for **13a** pro-*R* pose (0.9 kcal/mol) (Table S3). Since Phe56 was mutated to Ile in Phe56Ile/Trp288Ala, the stacking interaction between the phenyl (or substituted phenyl) ring and Phe56 was eliminated (Figs. S5 and S6). Consequently, ligand-strain energies in Phe56Ile/Trp288Ala were lower than those in Trp288Ala, typically ranging from 0.4 to 2.8 kcal/mol (Table S4). Moreover, for the 4-substituted ligands, pro-*S* poses generally exhibited lower strain than pro-*R* poses (Table S4). For the 4-bromo-substituted ligand (**7a**), standard docking simulation failed to obtain a valid pro-*R* pose in both mutants, likely due to the bulkiness of the bromo group. Therefore, IFD was employed, for which strain energy could not be computed using the same procedure as a standard docking simulation.

## Discussion

**Fig. 3:**
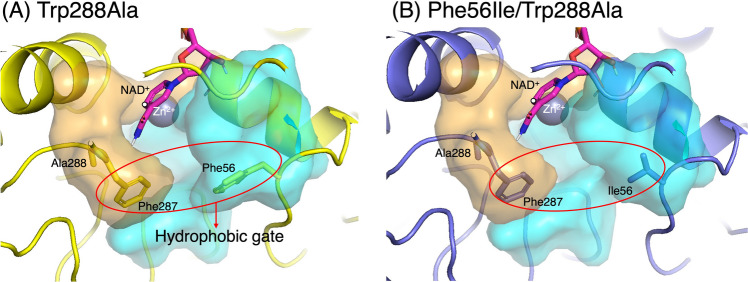
Binding pocket illustration of *Gc*APRD mutants: (**A**) Trp288Ala and (**B**) Phe56Ile/Trp288Ala (orange area: small pocket; blue area: large pocket; pink stick: NAD(H); white circle: C4 of NAD(H); gray sphere: catalytic zinc)

The reduction of benzophenone (**1a**) failed with the *Gc*APRD wild type but proceeded in high yield (≥ 80%) with Trp288Ala and Phe56Ile/Trp288Ala (Table 1). To investigate these improvements, the substrate binding site of *Gc*APRD was analyzed, and docking simulations were performed. A previous study has shown that the substrate binding site of *Gc*APRD is composed of two pockets: a large pocket (Ser47, His50, Ile51, Phe56, His66, Asn119, Leu122, Leu264) and a small pocket (Cys45, Asp156, Thr160, Phe287, Trp288) (Koesoema et al. 2019b). The two pockets in the mutants are shown in Fig. 3. Because of steric hindrance caused by Trp288 in the small pocket, the *Gc*APRD wild type yielded no poses of **1a** under the same docking simulation settings (Fig. S2). In contrast, the engineered small pocket of Trp288Ala was reported to be suitable for accommodating a phenyl ring (Koesoema et al. 2020a). Therefore, both Trp288Ala and Phe56Ile/Trp288Ala exhibited catalytic activity toward **1a**. The docking simulation results were also consistent with the experimental results (Tables S3 and S4). However, both mutants exhibited almost no reactivity toward 2-substituted substrates (**2a**, **5a**, **8a**, and **11a**). A similar phenomenon was also observed using *Lk*ADH and *Km*CR2, which showed lower conversions for the 2-substituted benzophenones than for 3- or 4-substituted ones (Li et al. 2019; Wu et al. 2020, 2021a).

In the Trp288Ala-catalyzed reduction of benzophenone analogs, (*R*)-alcohols were generally obtained for the reduction by 3-substituted substrates, but yields were low for the reduction of 4-substituted substrates (Figs. 2A and 2B). To elucidate the mechanism for the yield and enantioselectivity, the binding poses were investigated by docking simulations. In Trp288Ala, Phe56 and Phe287 formed a “hydrophobic gate” at the entrance between the large and small pockets (Fig. 3A). The phenyl or substituted phenyl ring of the ligand in the pro-*R* and pro-*S* poses, respectively, was located between these two residues, forming π–π stacking interaction (Figs. S3 and S4). Due to the steric hindrance caused by the phenyl or substituted phenyl ring and the “hydrophobic gate”, most ligands except **13a** exhibited high strain energies (2.4–5.2 kcal/mol), leading to a structural change around the carbonyl group. The phenyl ring interacting with the “hydrophobic gate” can also be seen in Fig. S2. Moreover, in the pro-*R* poses, steric hindrance in the small pocket might limit productive binding and cause relatively low yields and enantioselectivities for the 4-substituted substrates (**4a**, **7a**, and **10a**) (Fig. S4), whereas the corresponding effect was less significant for the 3-substituted substrates (Fig. S3), consistent with high yields and the (*R*)-enantioselectivities of 3-substituted substrates (**3a**, **6a**, **9a**, and **12a**).

**Fig. 4:**
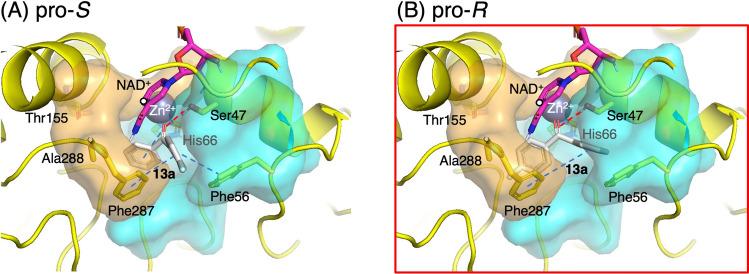
Comparison of pro-*S* and pro-*R* poses of **13a** in the docking model of *Gc*APRD Trp288Ala: (**A**) pro-*S* pose and (**B**) pro-*R* pose. The pose that match the experimental results is indicated with a red square (light-gray stick: ligand; pink stick: NAD(H); white circle: C4 of NADH; gray sphere: catalytic zinc; blue dashed line: π–π stacking; red dashed line: hydrogen bond)

For the reduction of **13a**, Trp288Ala exhibited the highest *R*-enantioselectivity (97% *ee*) among the reactions tested. Docking simulation indicated that the fluoro group was located close to the “hydrophobic gate” in the pro-*S* pose. The incompatibility between the hydrophilic fluoro group and “hydrophobic gate” prevented the formation of the pro-*S* pose and instead forced the 4-fluorophenyl ring into the small pocket, yielding a pro-*R* pose (Fig. 4). Moreover, in the resulting pro-*R* pose, the π–π stacking interaction with Phe56 was eliminated. As a result, the strain energy significantly decreased to 0.9 kcal/mol in the pro-*R* pose, whereas that in the pro-*S* pose was 3.9 kcal/mol (Table S3), indicating minimal structural change in the ligand and a more thermodynamically favorable binding mode in the pro-*R* pose, consistent with previous docking studies indicating that such highly strained conformations are unlikely to bind (Yang et al. 2021; Wallace et al. 2025). From these results, the significant ability of Trp288Ala to recognize the fluorine substituent was demonstrated, as hydrogen and fluoride have similar sizes and fluorine is substituted at the 4-position, far from the carbonyl group.

**Fig. 5:**
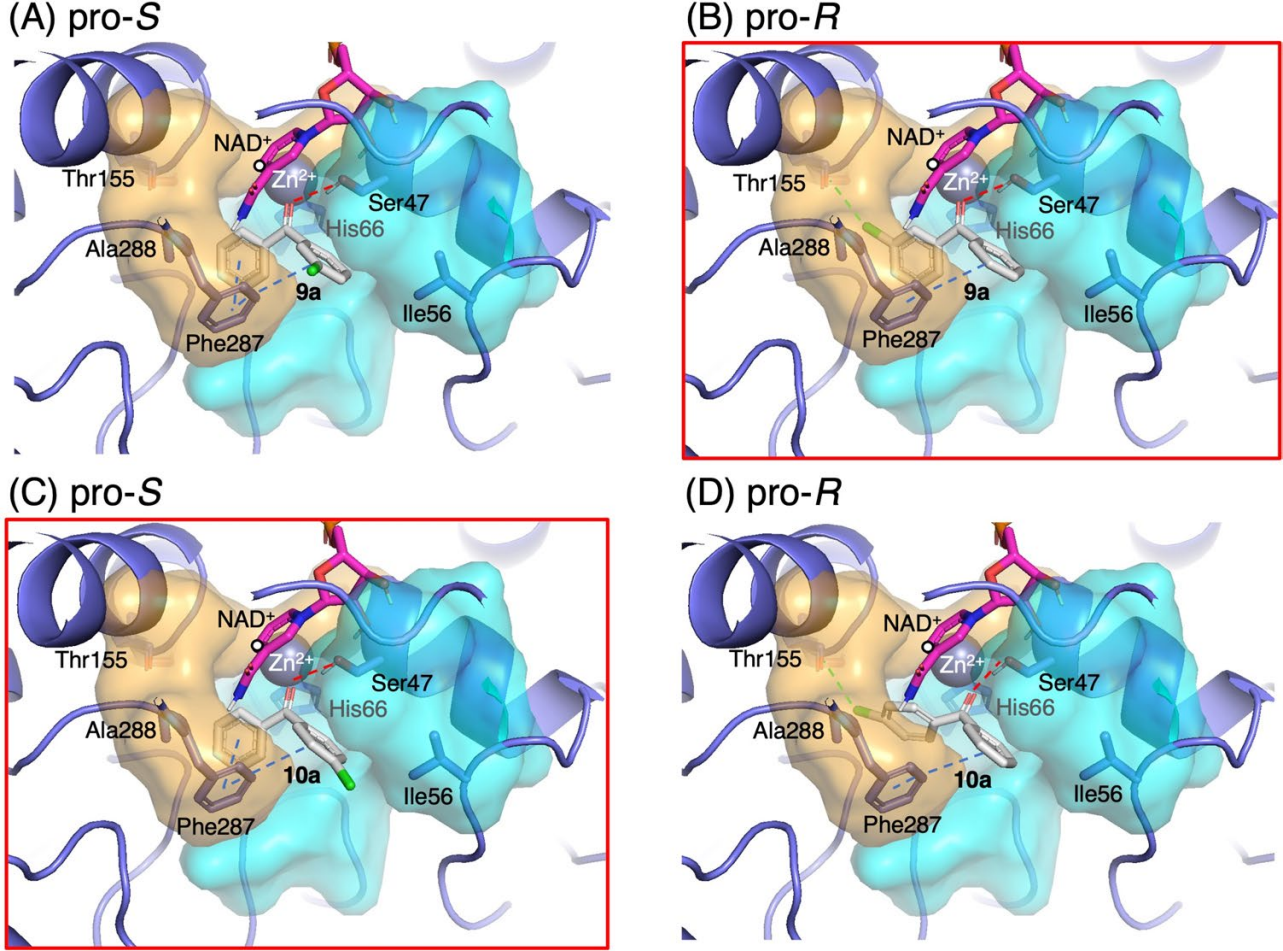
Comparison of pro-*S* and pro-*R* poses of **9a** and **10a** in the docking model of *Gc*APRD Phe56Ile/Trp288Ala: (**A**) pro-*S* pose of **9a**, (**B**) pro-*R* pose of **9a**, (**C**) pro-*S* pose of **10a**, and (**D**) pro-*R* pose of **10a**. The poses that match the experimental results are indicated with red squares (light-gray stick: ligand; pink stick: NAD(H); white circle: C4 of NADH; gray sphere: catalytic zinc; blue dashed line: π–π stacking; red dashed line: hydrogen bond; green dashed line: halogen bond)

In contrast to Trp288Ala, Phe56Ile/Trp288Ala generally showed (*R*)-enantioselectivity toward 3-substituted substrates and (*S*)-enantioselectivity toward 4-substituted substrates (Fig. 2D). The simulation results of Phe56Ile/Trp288Ala indicated that the pocket entrance was widened, and the π–π stacking interaction between the ligand and Phe56 was eliminated (Fig. 2B and Figs. S5 and S6). As a result, the ligand showed increased flexibility in the pockets. For 3-substituted ligands, in the pro-*S* pose, the substituent was near the pocket edge with only a few residues within 5 Å of it. Therefore, it exhibited high solvent exposure, which could unstabilize binding as reported in the binding pose metadynamics study (Fusani et al. 2020). In the pro-*R* pose, the substituted phenyl ring was stabilized by interactions with residues in the small pocket (Fig. S5). For example, the 3-methylphenyl ring of **3a** was stabilized by hydrophobic interactions (Fig. S5B). The 3-bromophenyl ring of **6a** formed a π–π stacking interaction with His66 and a halogen bond with Thr155. A halogen bond was also observed between the chloro group and Thr155 in the pro-*R* pose of **9a** (Fig. 5). These interactions may have contributed to the high enantioselectivity as reported in the case of *Kp*ADH-catalyzed reduction of diaryl ketones (Xu et al. 2018). Therefore, high (*R*)-enantioselectivities were observed for the Phe56Ile/Trp288Ala-catalyzed reduction of 3-substituted substrates: 89% *ee* for (*R*)-**3b**, 87% *ee* for (*R*)-**6b,** and 85% *ee* for (*R*)-**9b**.

Moreover, due to the loss of the “hydrophobic gate” at the pocket entrance in Phe56Ile/Trp288Ala, the ligands generally exhibited lower strain energy during the binding. The low strain energies indicated that the ligands entered the binding site with minimal structural change, making the resulting pose more thermodynamically stable (Yang et al. 2021; Wallace et al. 2025). Particularly for the 4-substituted ligands, the pro-*S* pose showed lower strain energy than the pro-*R* pose, resulting in a (*S*)-enantiopreference (Table S4). Additionally, compared with the 3-substituted ligands, the entry of the 4-substituted ligands to form a pro-*R* pose was limited by steric hindrance in the small pocket, as also observed in Trp288Ala. For example, in the docking of **4a**, the strain energies of the pro-*S* and pro-*R* poses were 1.6 and 3.9 kcal/mol, respectively. Similarly, for **10a**, the strain energies of the pro-*S* and pro-*R* poses were 1.5 kcal/mol and 6.3 kcal/mol, respectively. Consistently, the experimental data showed high (*S*)-enantioselectivity toward **4a** and **10a** with 88% and 81% *ee*, respectively.

Based on these results, the combined effects of pocket structural changes, ligand–residue interactions, and low ligand strain result in a clear enantiopreference of Phe56Ile/Trp288Ala toward benzophenone analogs. It is worth noting that the benzophenone analogs are more structurally symmetrical than the previously studied acetophenones and 2-benzoylpyridines (Koesoema et al. 2020a; Tang et al. 2024b), so the enzyme encountered more difficulties in discriminating between the pro-*S* and pro-*R* poses, but these were overcame by using the engineered *Gc*APRDs.

Moreover, this study investigating the engineered *Gc*APRDs successfully broadened the known catalytic scope of enzymes from the MDR family toward various substituted diaryl ketones, while the only previously reported MDR is restricted to reducing a few benzophenones including **10a** (Liu et al. 2019; Qu et al. 2019) (Table S5). In contrast, *Gc*APRD mutants can additionally reduce multiple 3- and 4-substituted benzophenones, representing the first demonstration of such activity in the MDR family. Meanwhile, a few enzymes from the SDR family have been reported to reduce some of the substrates used in this study, as listed in Table S5. Notably, the yields and enantioselectivities of the reductions catalyzed by *Gc*APRD mutants were much higher than those by the SDRs depending on the kind of substrates. Particularly, *Gc*APRD Trp288Ala exhibited the highest (*R*)-enantioselectivity (97% *ee*) in the reduction of 4-fluorobenzophenone (**13a**) due to the presence of “hydrophobic gate” and the differences in strain energies between pro-*S* and pro-*R* poses. On the other hand, *Kp*ADH mutant, *Lk*ADH mutant, and *Km*CR2 exhibited at most 61.7% *ee* (*R*), 40.3% *ee* (*R*), and 81% *ee* (*R*), respectively (Wang et al. 2018; Li et al. 2019; Wu et al. 2021a). In addition, *Gc*APRD Phe56Ile/Trp288Ala also exhibited higher (*R*)-enantioselectivity (89% *ee*) toward 3-methylbenzophenone (**3a**) than *Lk*ADH mutant (54% *ee* (*R*)) and *Km*CR2 (74% *ee* (*R*)) (Li et al. 2019; Wu et al. 2021a).

In conclusion, the challenging benzophenone analogs, particularly the 3- and 4-substituted substrates, were successfully reduced by both *Gc*APRD Trp288Ala and Phe56Ile/Trp288Ala. Trp288Ala generally produced (*R*)-alcohols following anti-Prelog’s rule, reaching 97% *ee* for the reduction of 4-fluorobenzophenone (**13a**), indicating effective discrimination between the two groups adjacent to the carbonyl group in this structurally highly symmetric ketone. Compared with Trp288Ala, Phe56Ile/Trp288Ala exhibited higher reactivity and an interesting enantiopreference. It generally exhibited (*R*)-enantioselectivity toward 3-substituted substrates following anti-Prelog’s rule, but (*S*)-enantioselectivity toward 4-substituted substrates following Prelog’s rule. Both (*R*)- and (*S*)-alcohols were generally obtained with high *ee* values exceeding 80%. Phe56Ile/Trp288Ala also maintained similarly high yields and enantioselectivity under the scaled-up conditions, providing a promising biocatalytic method for the sustainable synthesis of chiral diaryl methanols.

## Supplementary Information

Below is the link to the electronic supplementary material.

ESM 1 (PDF 15.1 MB)

ESM 2 (PDF 3.23 MB)

## Data Availability

Supplementary materials and appendix (1H-NMR and HPLC spectra) related to this article can be found.
