# Supplementary material for: Highly enantioselective reduction of benzophenones by engineered Geotrichum candidum alcohol dehydrogenase
Source: Appl Microbiol Biotechnol. 2026 Jan 29;110(1):48. doi: 10.1007/s00253-026-13717-0 (PMC12858457; doi:10.1007/s00253-026-13717-0)
Supplement: Supplementary file 1 — (PDF 15.1 MB) [file 253_2026_13717_MOESM1_ESM.pdf]

Journal name: Applied Microbiology and Biotechnology

Appendix

Highly Enantioselective Reduction of Benzophenones by Engineered *Geotrichum candidum* Alcohol Dehydrogenase

Zhongyao Tang[1], Guillermo Germán Otárola Tejada[1,2], Afifa Ayu Koesoema[1], Tomoko Matsuda[1*]

1. Department of Life Science and Technology, School of Life Science and Technology, Institute of Science Tokyo, 4259 Nagatsuta-cho Midori-ku, Yokohama 226-8501, Japan
2. Department of Organic and Inorganic Chemistry, University of Alcalá, Ctra. Madrid-Barcelona km 33.100, Alcalá de Henares, Madrid 28805, Spain

Corresponding author:
Tomoko Matsuda (tmatsuda@bio.titech.ac.jp, +81-45-924-5757)

Table of contents

1. ^{1}H-NMR spectra of standard racemic alcohols, *rac*-**3b**, *rac*-**6b**, *rac*-**12b**, and *rac*-**13b**............1

2. HPLC chromatograms of standard racemic alcohols and products from *Gc*APRD mutants-catalyzed reductions 3

3. ^{1}H-NMR spectra of products from *Gc*APRD Trp288Ala-catalyzed reductions 11

4. ^{1}H-NMR spectra of products from *Gc*APRD Phe56Ile/Trp288Ala-catalyzed reductions.......14

5. ^{1}H-NMR spectra and HPLC chromatograms of products from *Gc*APRD Phe56Ile/Trp288Ala-catalyzed scaled-up reductions..19

1. ^{1}H-NMR spectra of standard racemic alcohols, *rac*-3b, *rac*-6b, *rac*-12b, and *rac*-13b

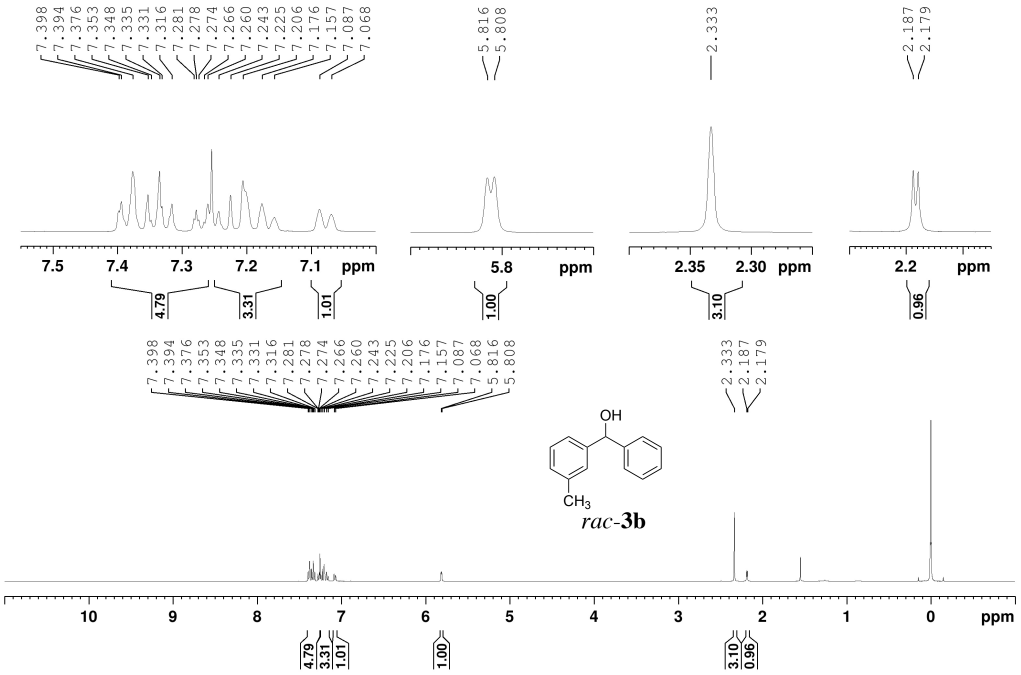

^{1}H-NMR spectrum of *rac*-**3b**

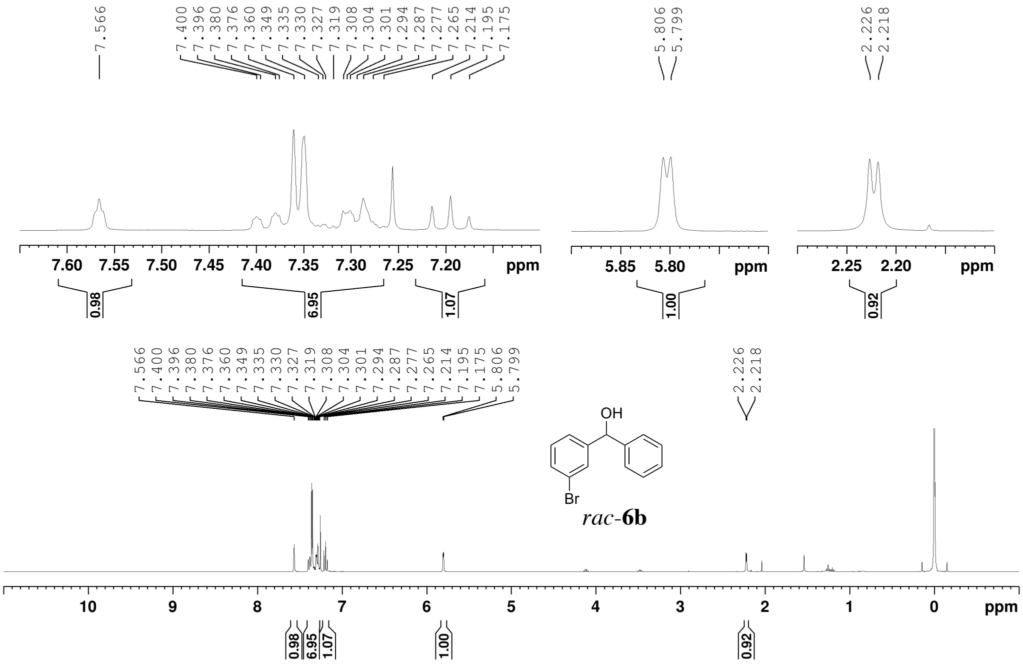

^{1}H-NMR spectrum of *rac*-**6b**

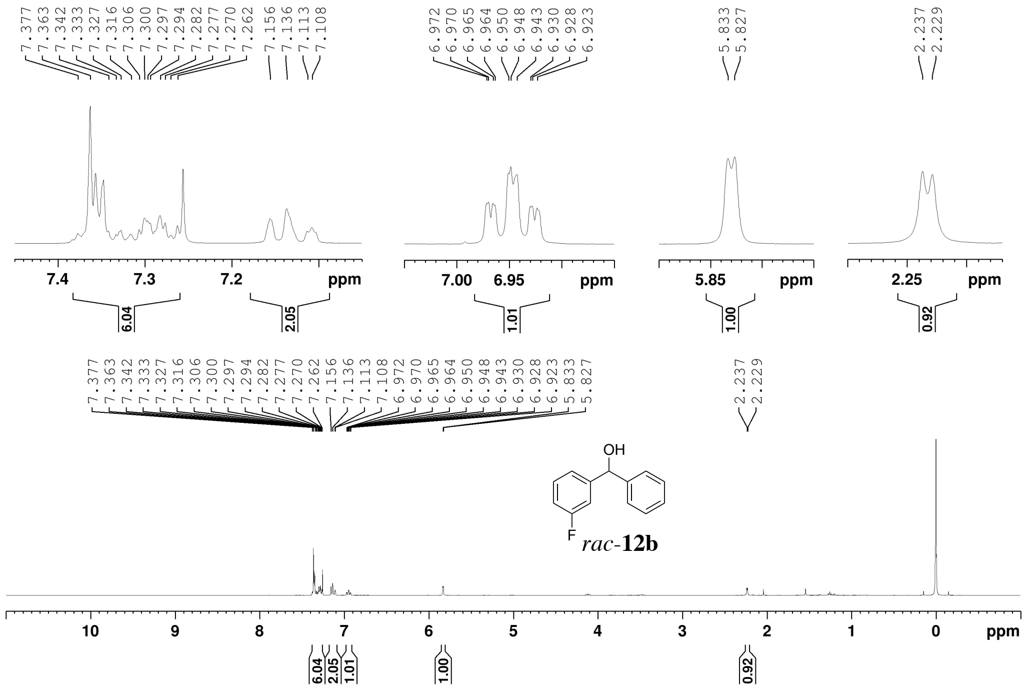

^{1}H-NMR spectrum of *rac*-**12b**

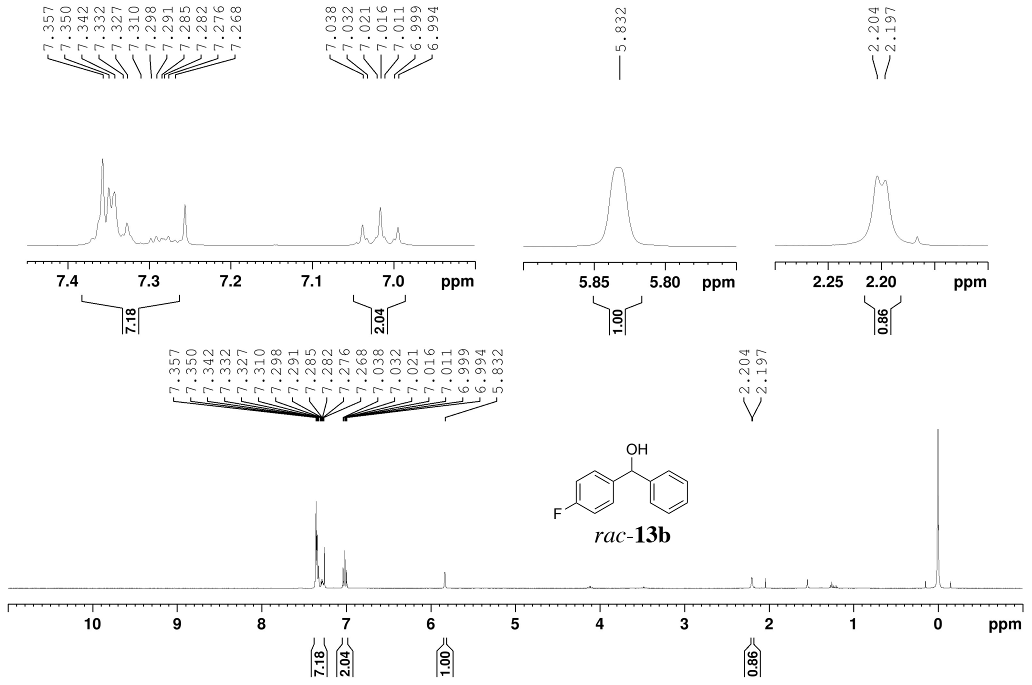

^{1}H-NMR spectrum of *rac*-**13b**

2. HPLC chromatograms of standard racemic alcohols and products from *Gc*APRD mutants-catalyzed reductions

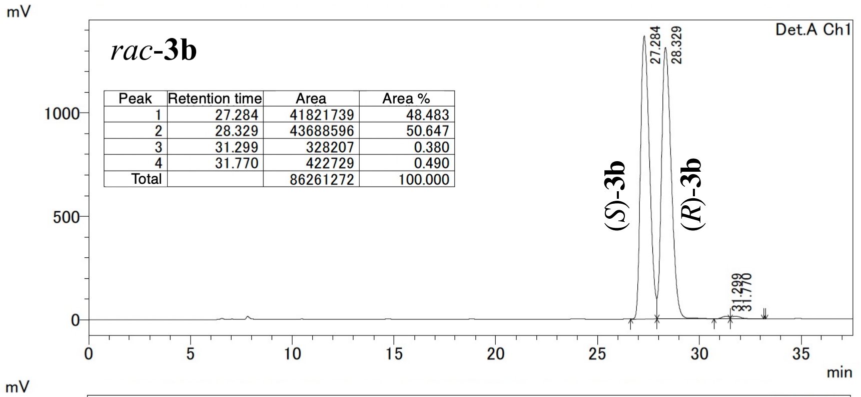

Peak	Retention time	Area	Area %
1	27.284	41821739	48.483
2	28.329	43688596	50.647
3	31.299	328207	0.380
4	31.770	422729	0.490
Total		86261272	100.000

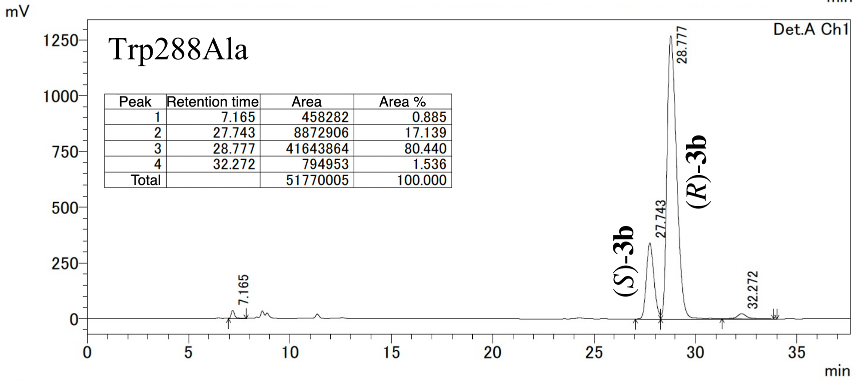

Peak	Retention time	Area	Area %
1	7.165	458282	0.885
2	27.743	8872906	17.139
3	28.777	41643864	80.440
4	32.272	794953	1.536
Total		51770005	100.000

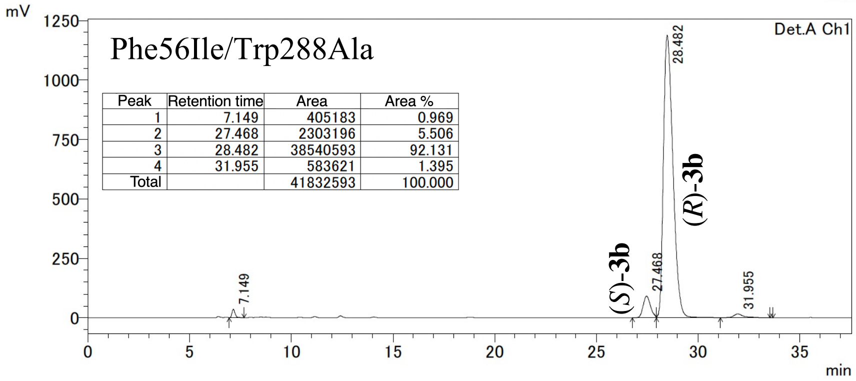

Peak	Retention time	Area	Area %
1	7.149	405183	0.969
2	27.468	2303196	5.506
3	28.482	38540593	92.131
4	31.955	583621	1.395
Total		41832593	100.000

HPLC chromatograms of *rac*-**3b** and products from *Gc*APRD mutants-catalyzed reduction of **3a**

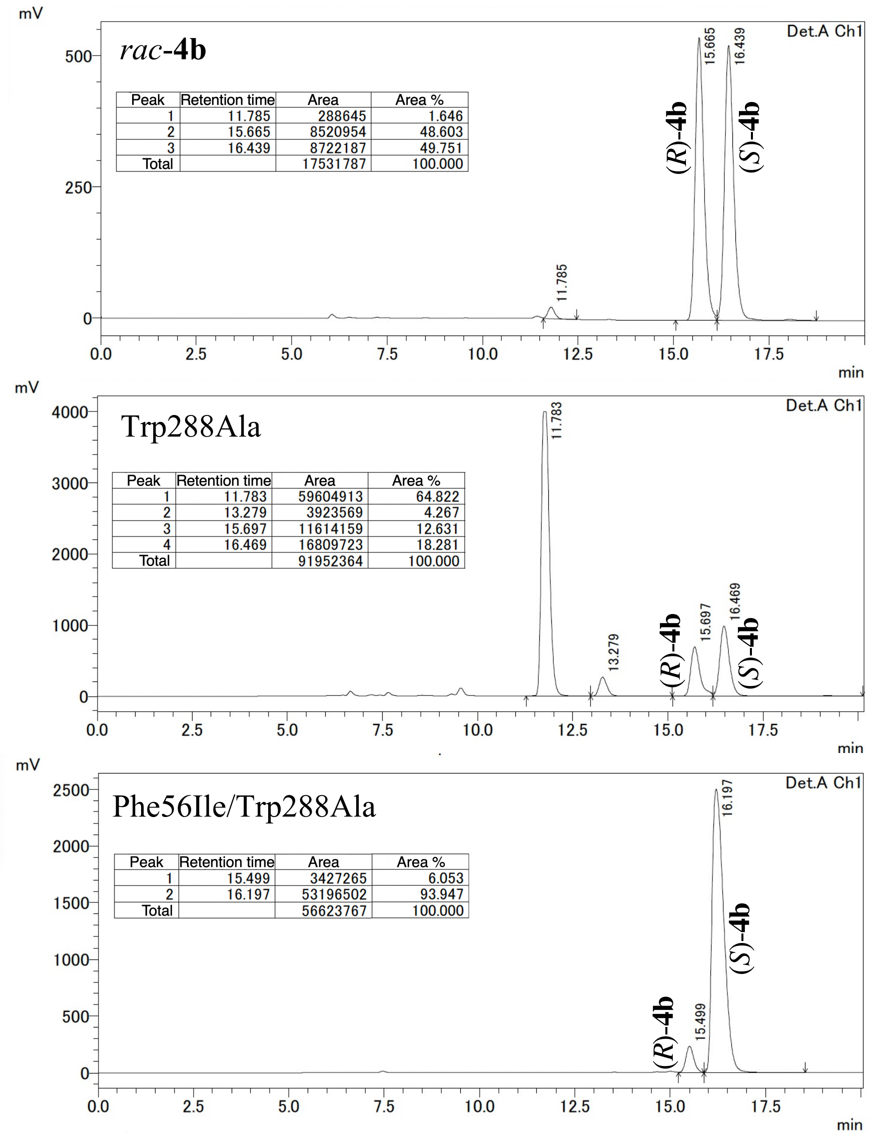

HPLC chromatograms of *rac*-**4b** and products from *Gc*APRD mutants-catalyzed reduction of **4a**

The sample of Trp288Ala was taken directly from the crude reaction mixture.

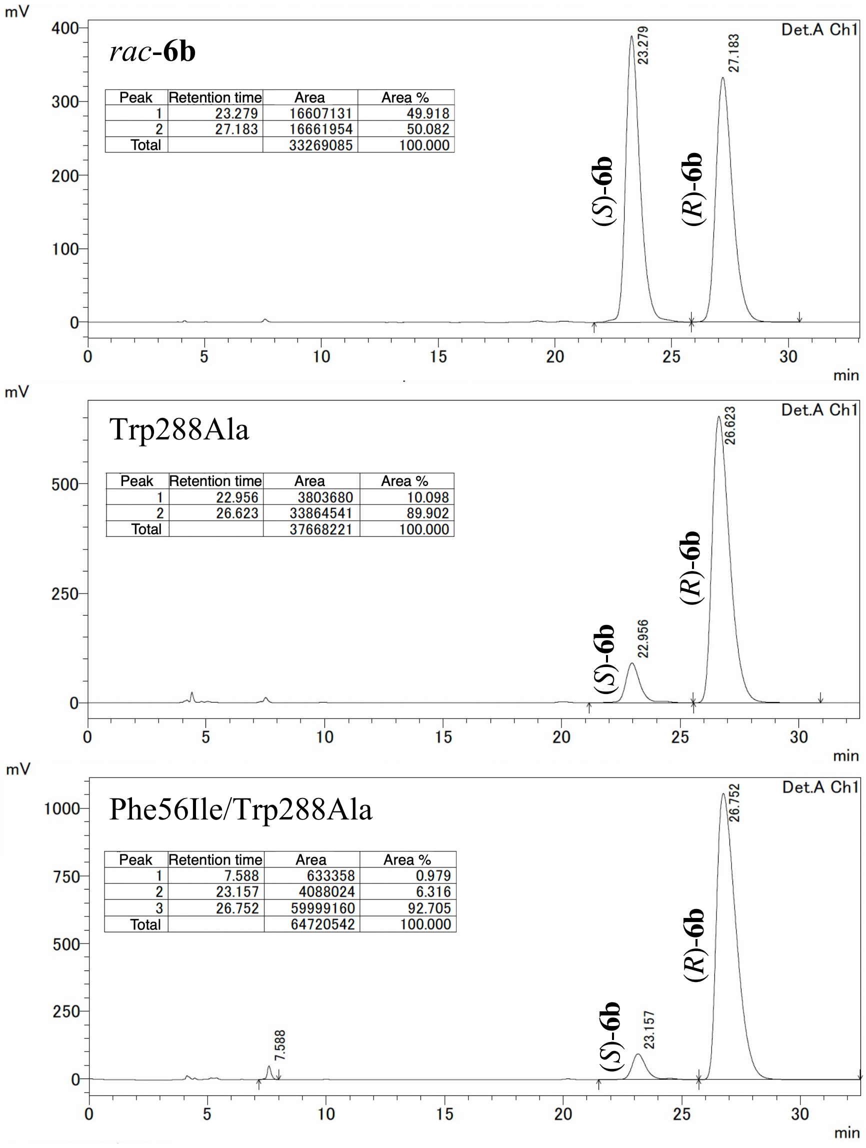

HPLC chromatograms of *rac*-**6b** and products from *Gc*APRD mutants-catalyzed reduction of **6a**

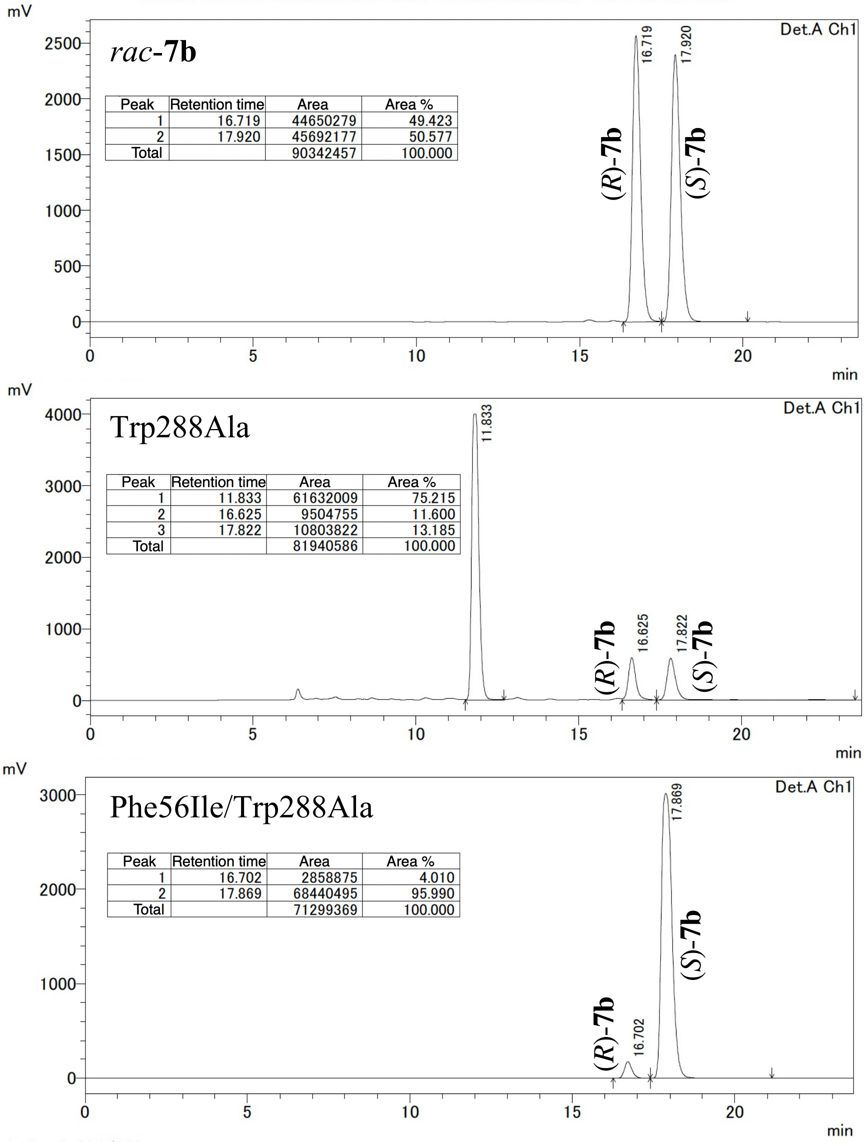

HPLC chromatograms of *rac*-**7b** and products from *Gc*APRD mutants-catalyzed reduction of **7a**

The sample of Trp288Ala was taken directly from the crude reaction mixture.

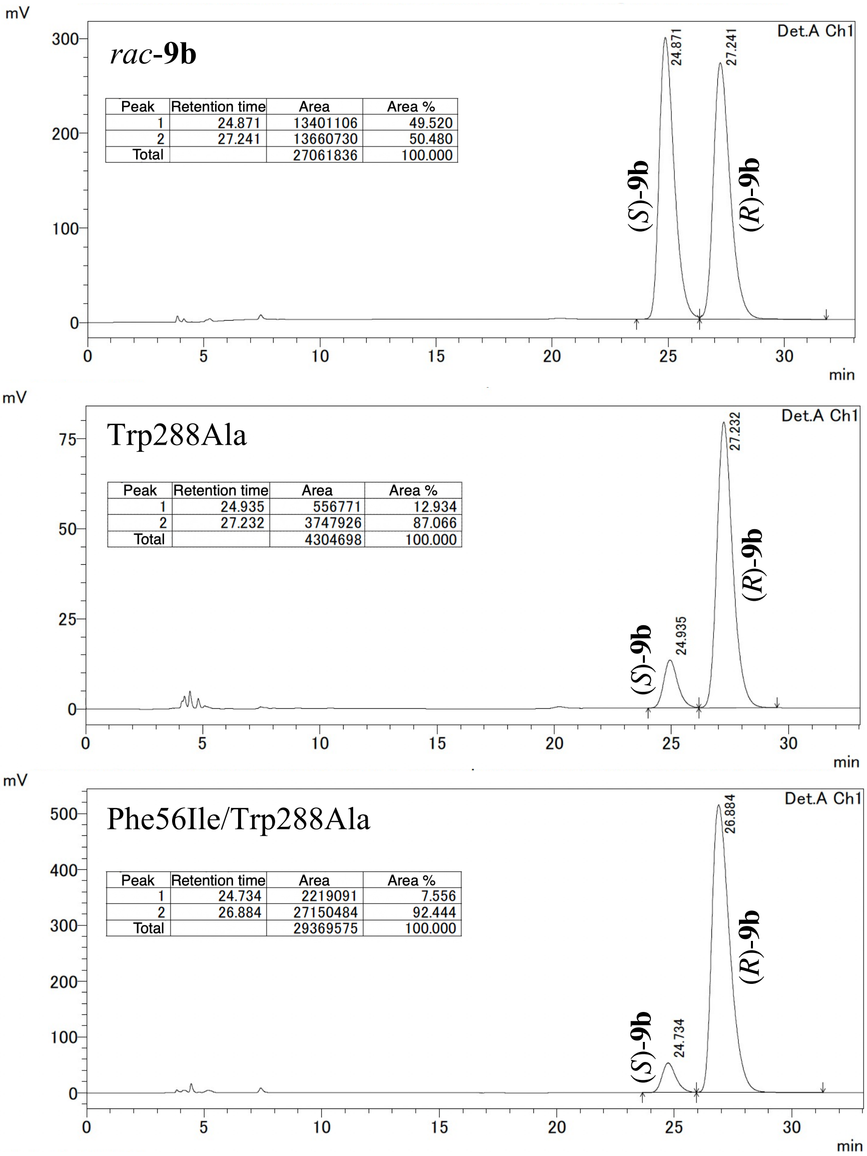

HPLC chromatograms of *rac*-**9b** and products from *Gc*APRD mutants-catalyzed reduction of **9a**

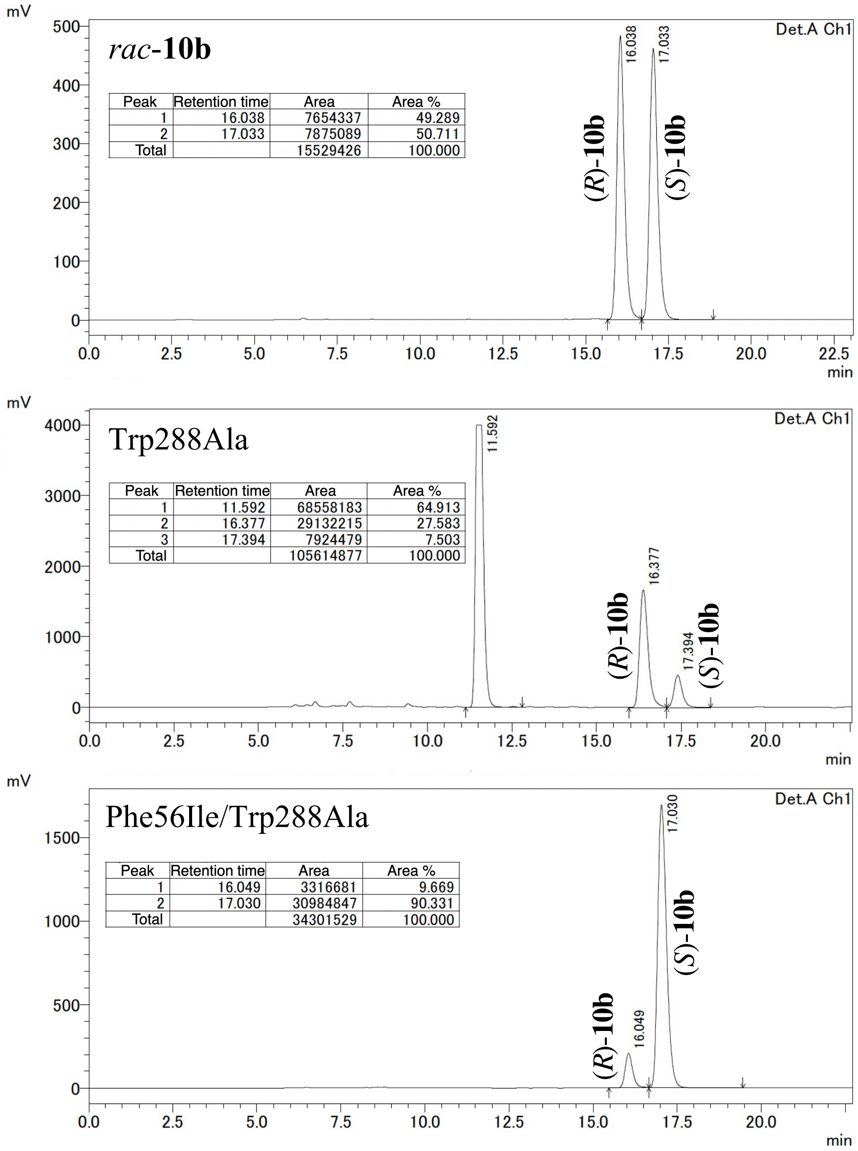

HPLC chromatograms of *rac*-**10b** and products from *Gc*APRD mutants-catalyzed reduction of **10a**

The sample of Trp288Ala was taken directly from the crude reaction mixture.

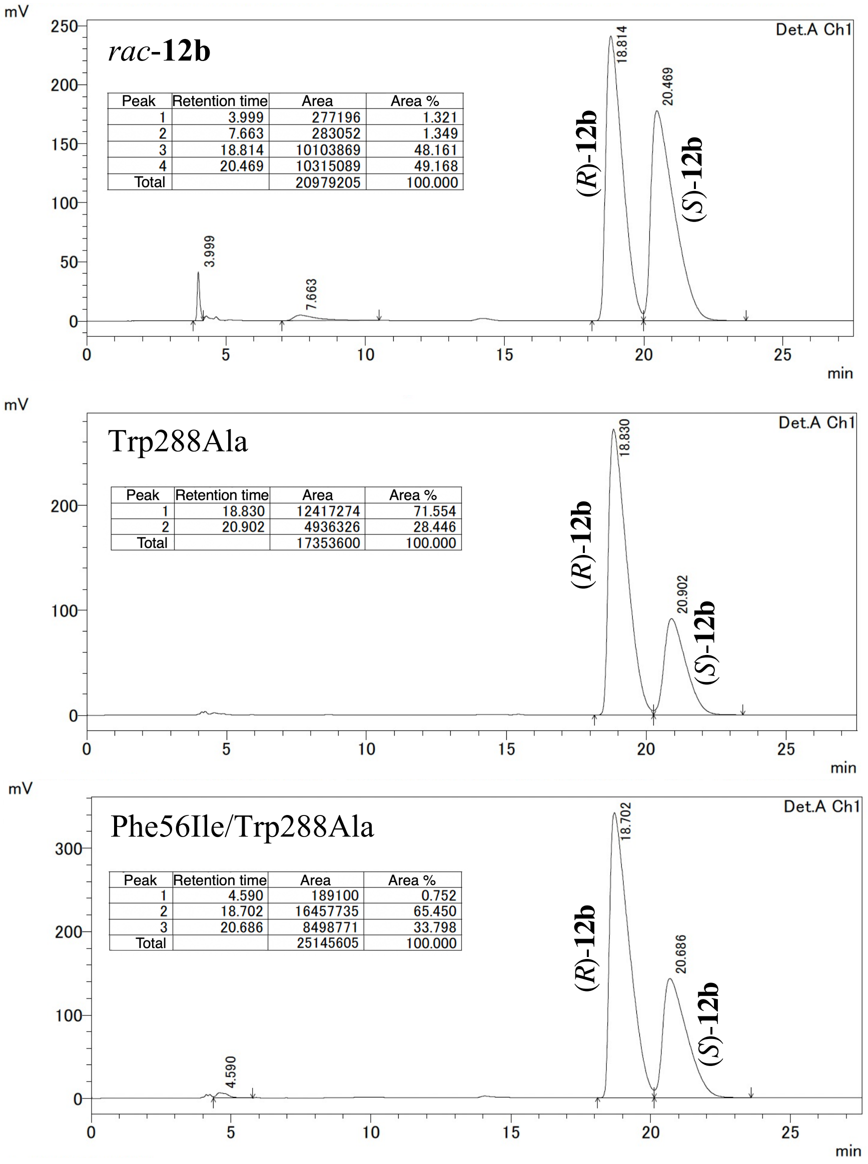

HPLC chromatograms of *rac*-**12b** and products from *Gc*APRD mutants-catalyzed reduction of **12a**

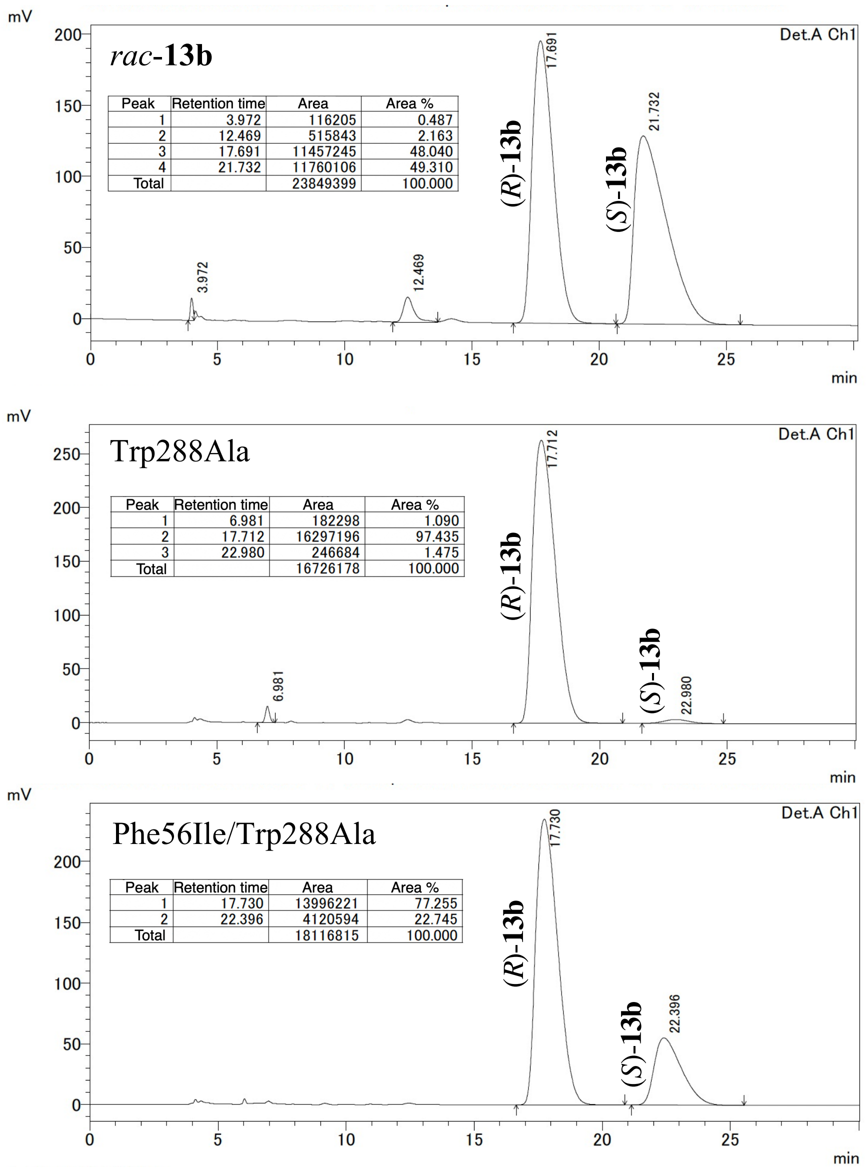

HPLC chromatograms of *rac*-**13b** and products from *Gc*APRD mutants-catalyzed reduction of **13a**

3. ¹H-NMR spectra of products from *Gc*APRD Trp288Ala-catalyzed reductions

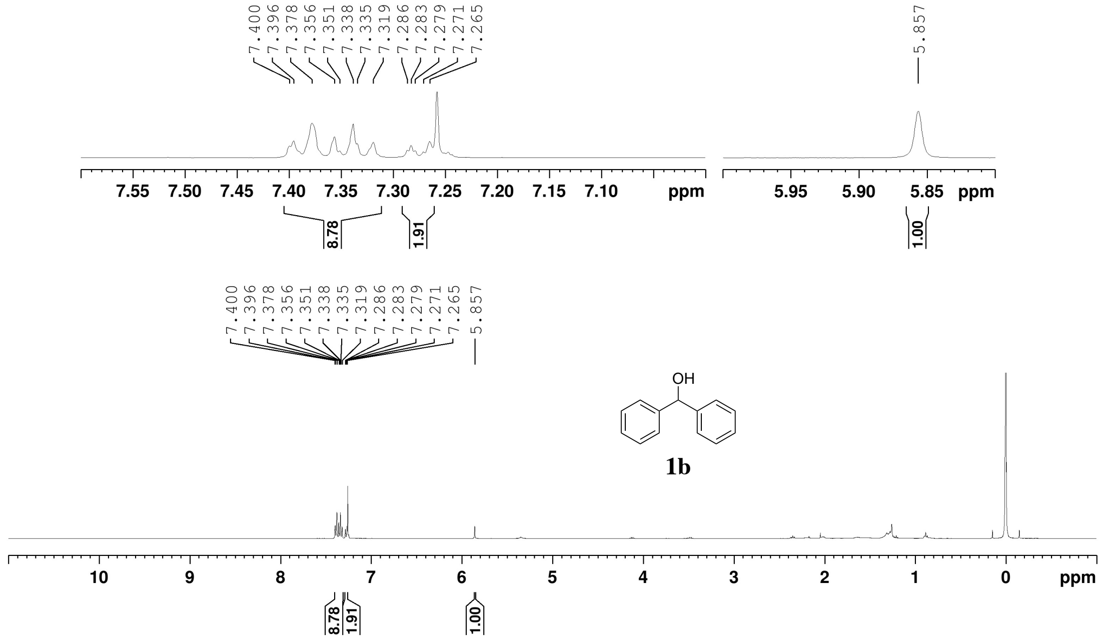

¹H-NMR spectrum of product from *Gc*APRD Trp288Ala-catalyzed reduction of **1a**

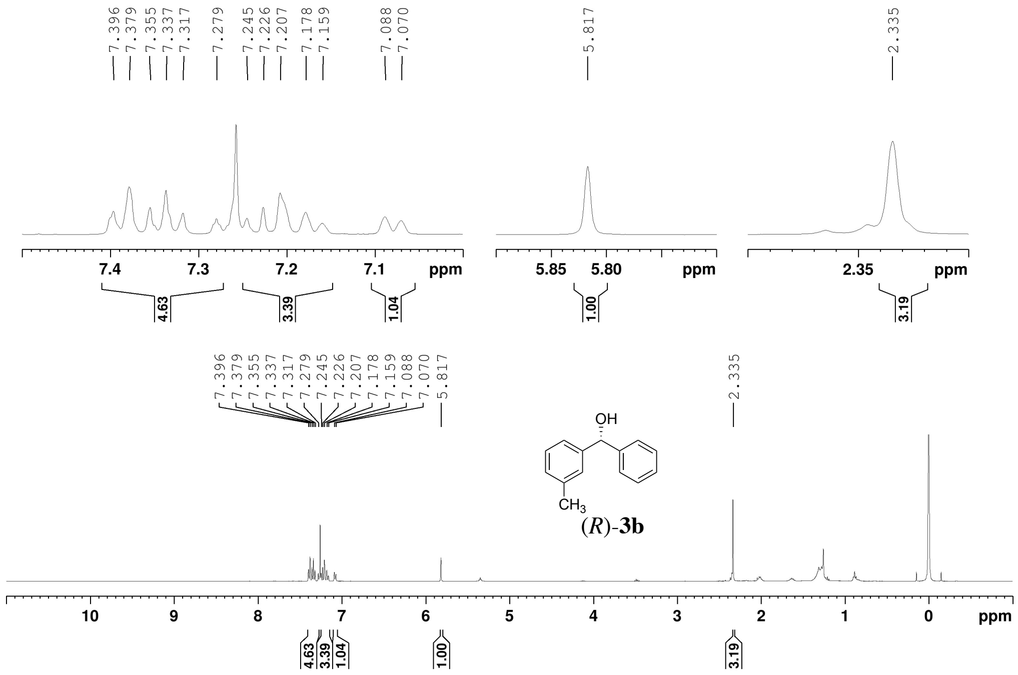

¹H-NMR spectrum of product from *Gc*APRD Trp288Ala-catalyzed reduction of **3a**

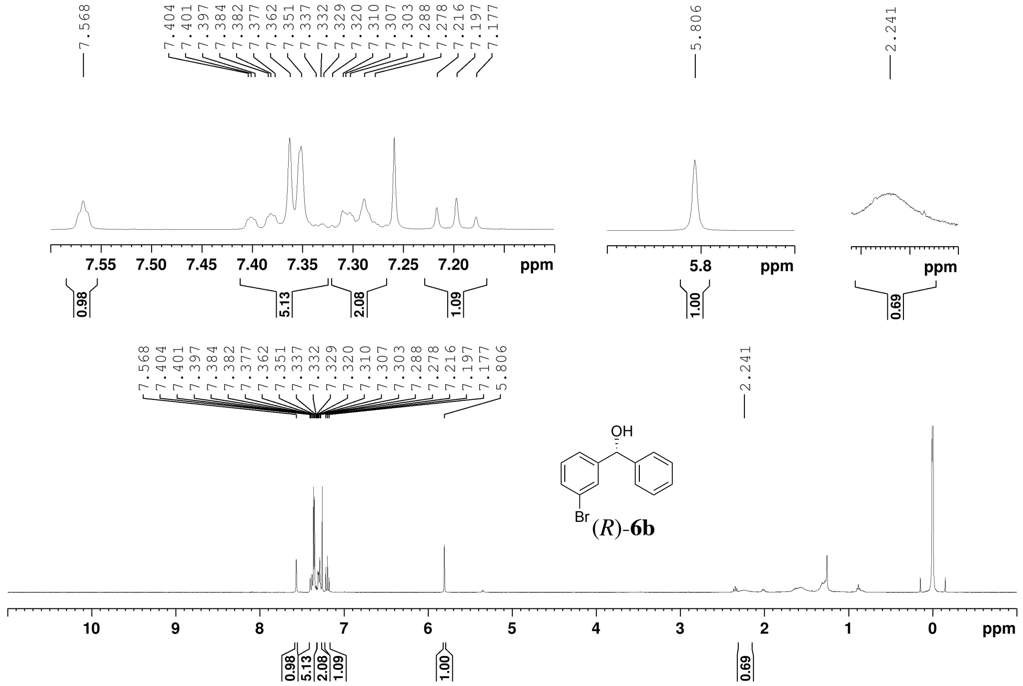

^{1}H-NMR spectrum of product from *Gc*APRD Trp288Ala-catalyzed reduction of **6a**

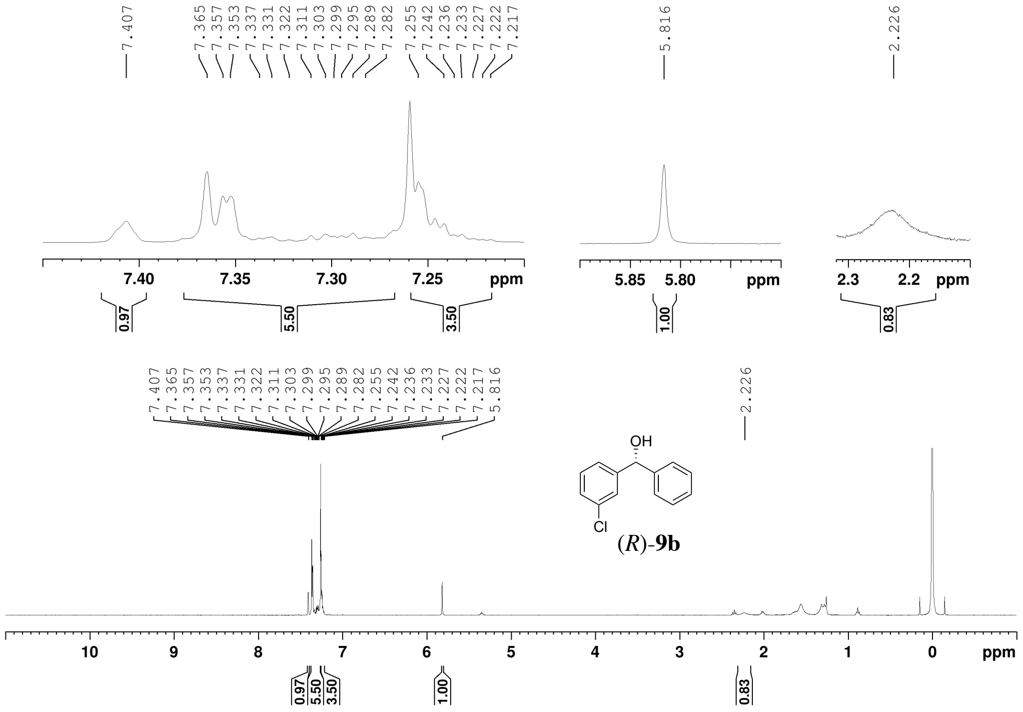

^{1}H-NMR spectrum of product from *Gc*APRD Trp288Ala-catalyzed reduction of **9a**

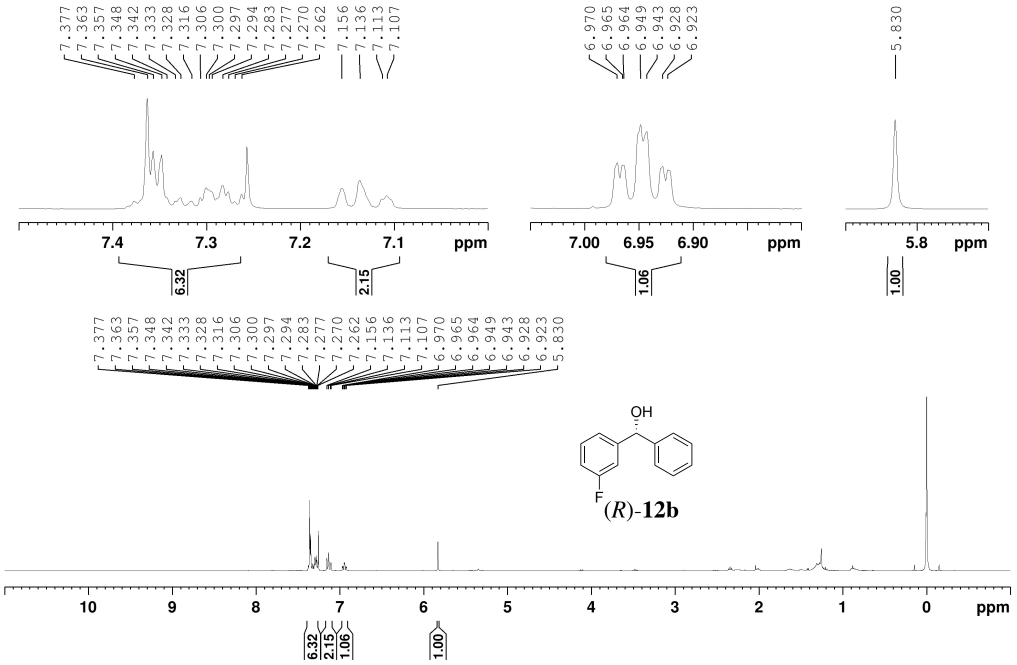

^{1}H-NMR spectrum of product from *Gc*APRD Trp288Ala-catalyzed reduction of **12a**

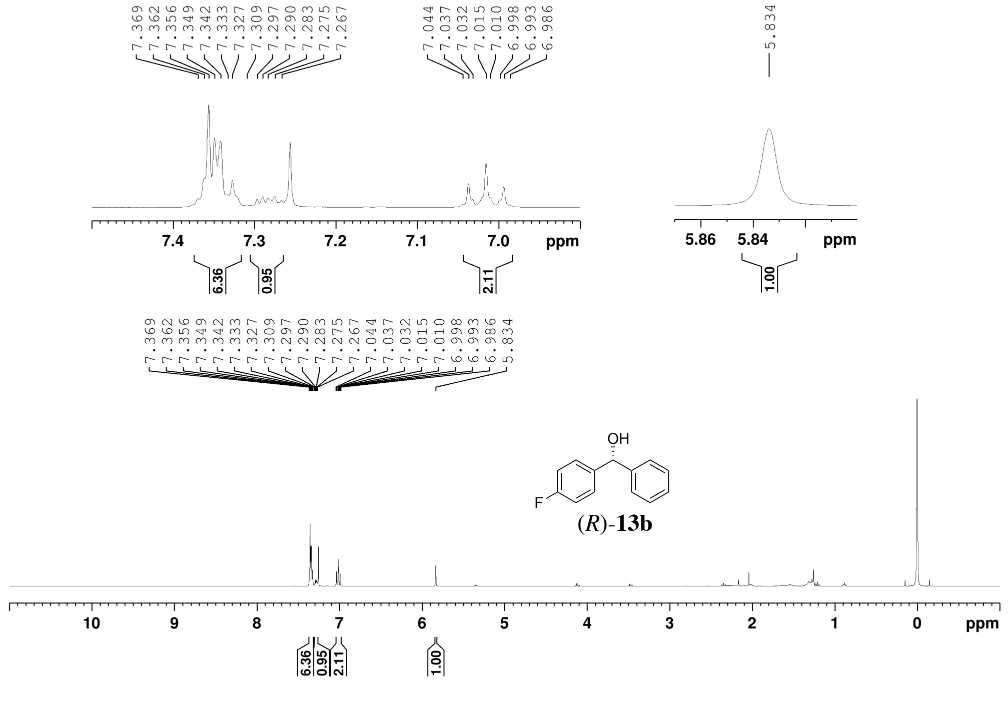

^{1}H-NMR spectrum of product from *Gc*APRD Trp288Ala-catalyzed reduction of **13a**

4. ^{1}H-NMR spectra of products from *Gc*APRD Phe56Ile/Trp288Ala-catalyzed reductions

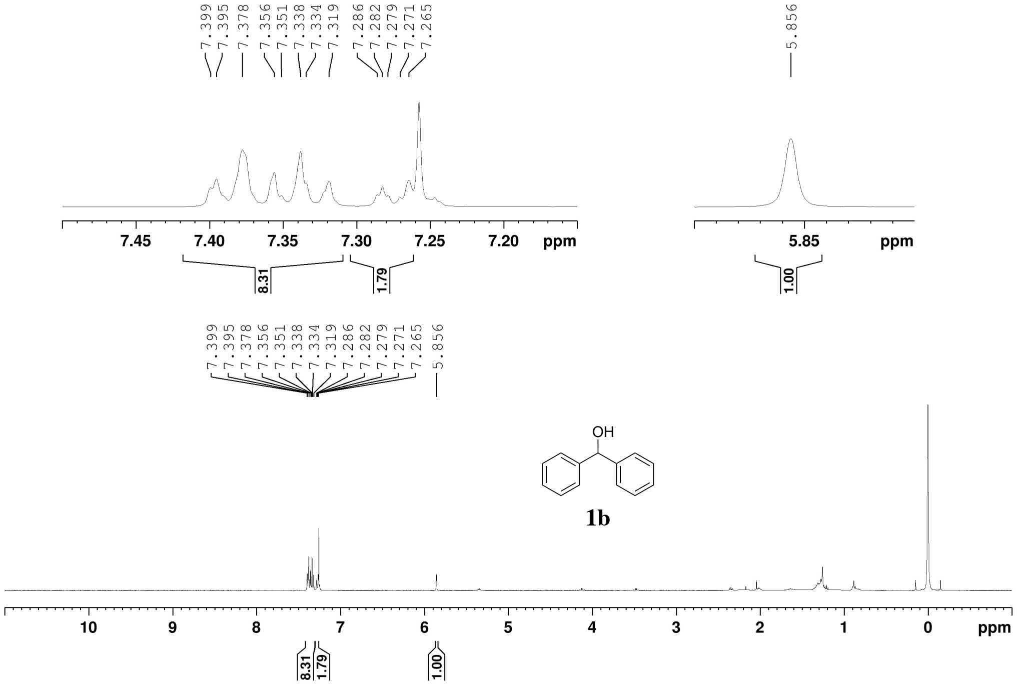

^{1}H-NMR spectrum of product from *Gc*APRD Phe56Ile/Trp288Ala-catalyzed reduction of **1a**

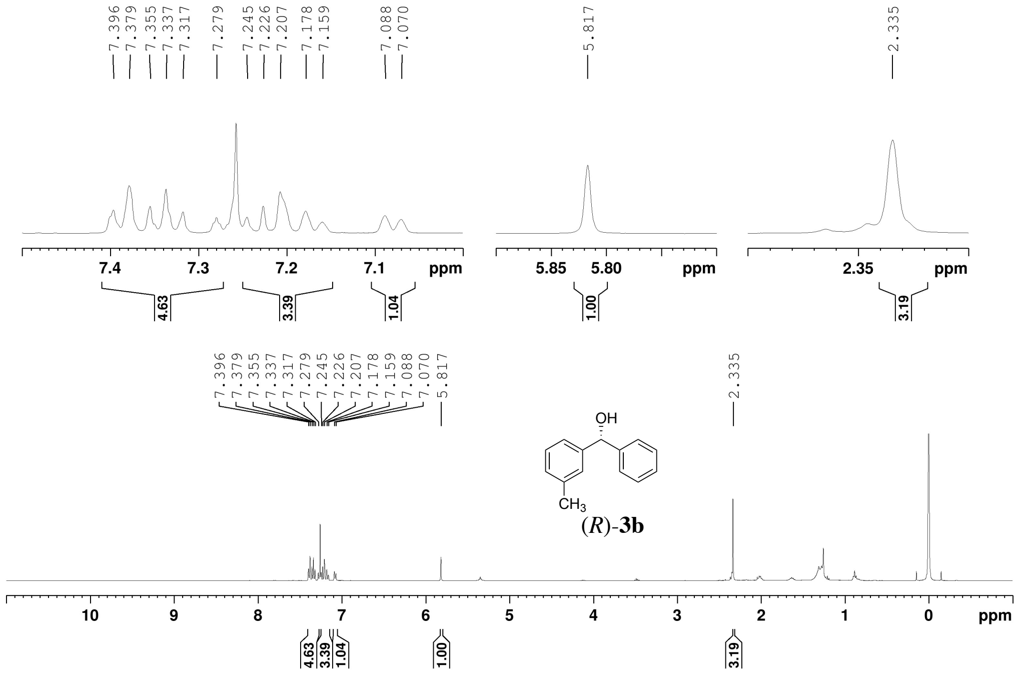

^{1}H-NMR spectrum of product from *Gc*APRD Phe56Ile/Trp288Ala-catalyzed reduction of **3a**

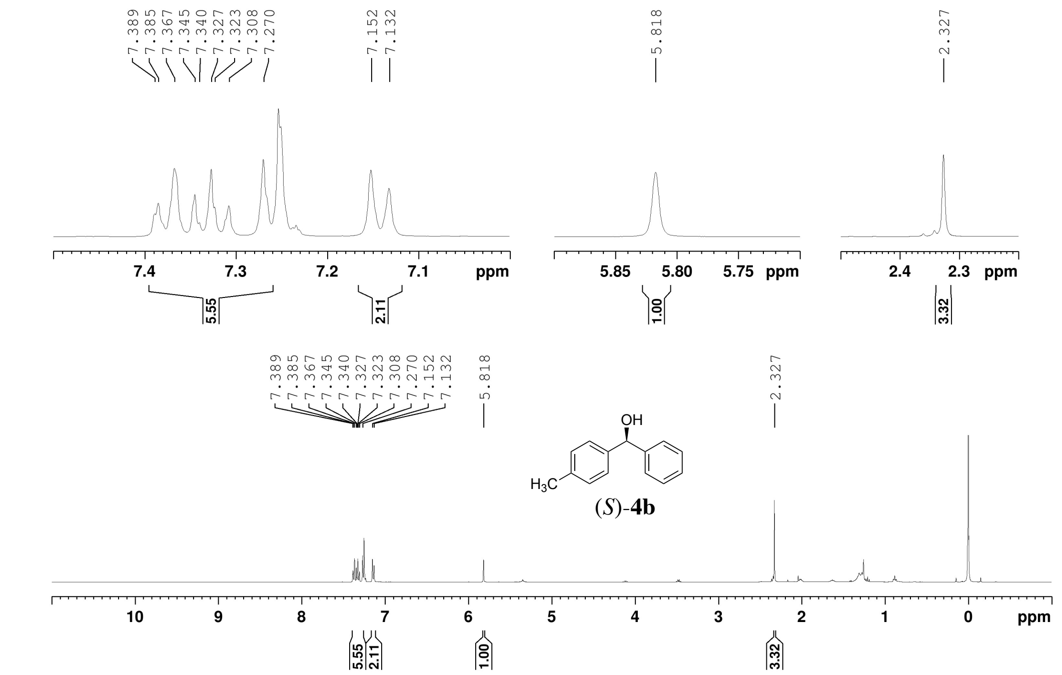

^{1}H-NMR spectrum of product from *Gc*APRD Phe56Ile/Trp288Ala-catalyzed reduction of **4a**

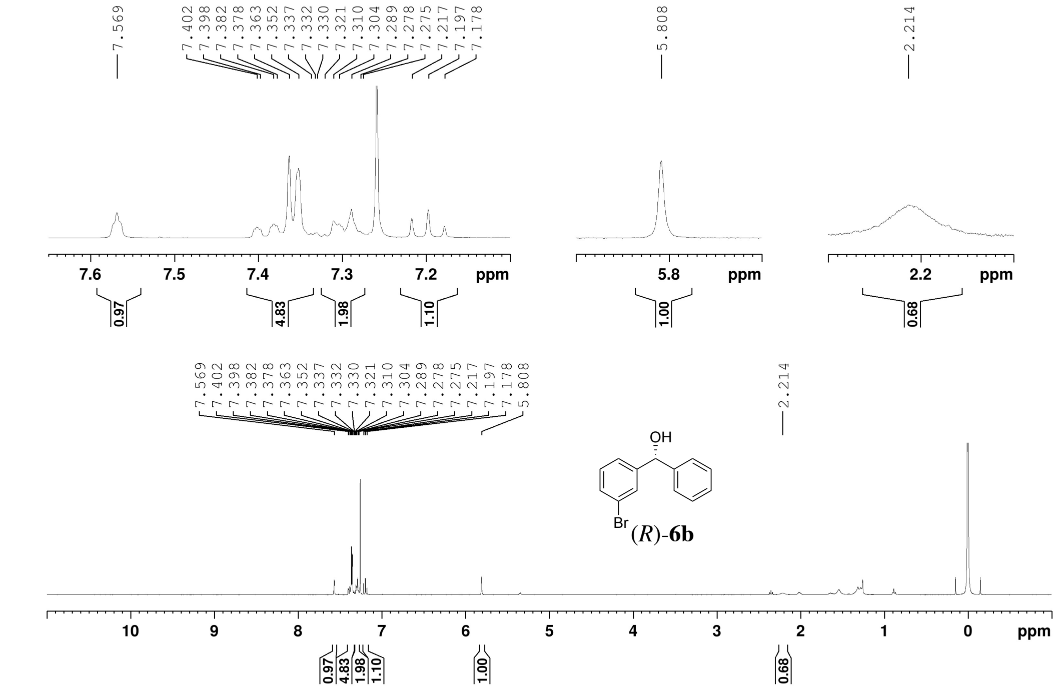

^{1}H-NMR spectrum of product from *Gc*APRD Phe56Ile/Trp288Ala-catalyzed reduction of **6a**

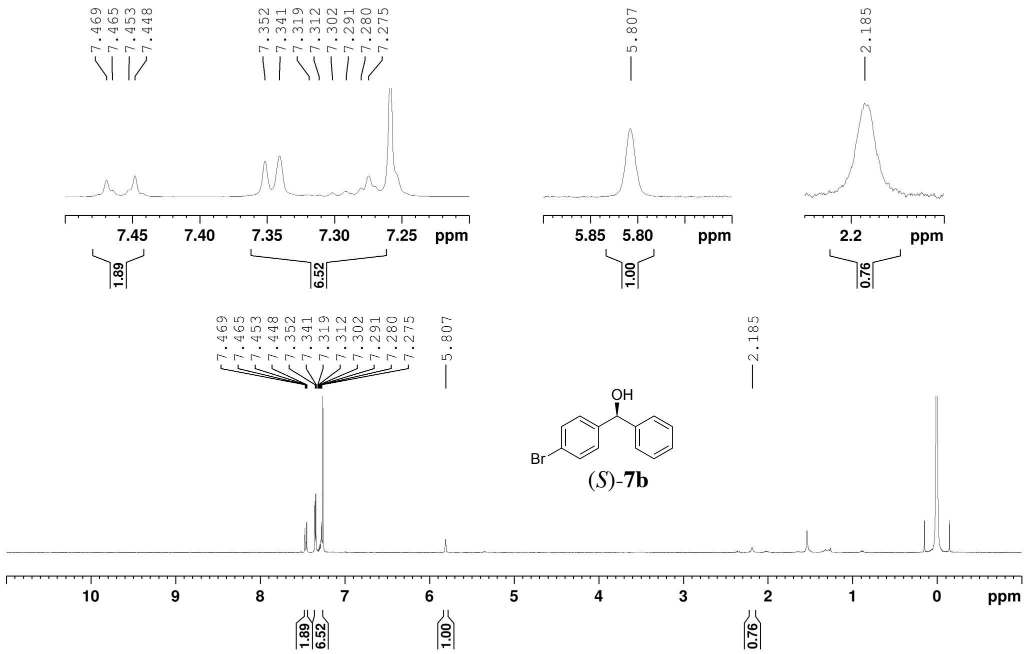

^{1}H-NMR spectrum of product from *Gc*APRD Phe56Ile/Trp288Ala-catalyzed reduction of **7a**

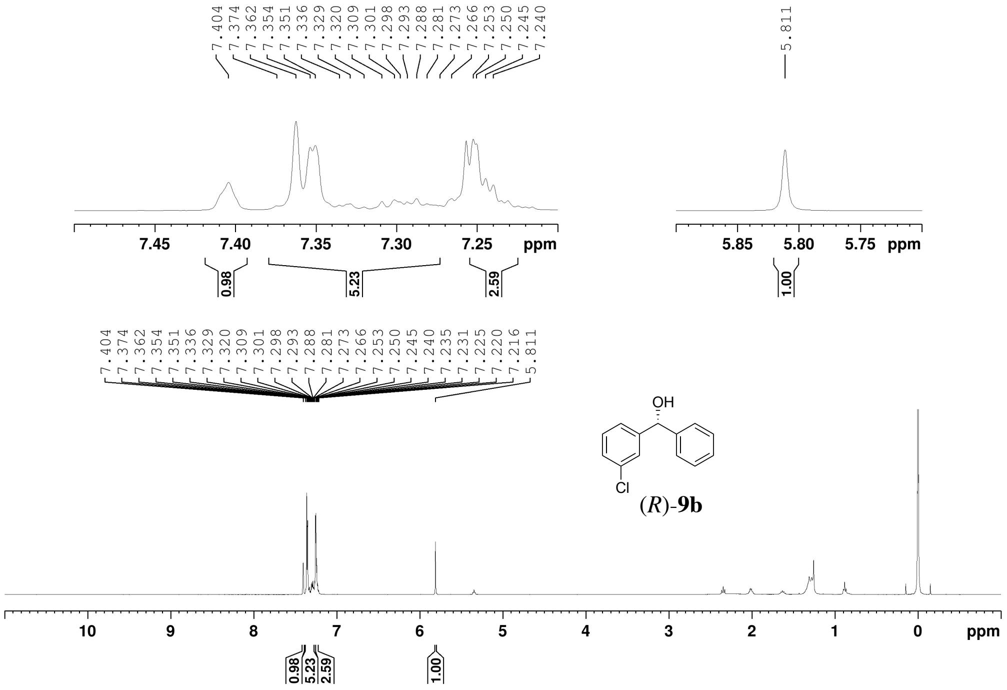

^{1}H-NMR spectrum of product from *Gc*APRD Phe56Ile/Trp288Ala-catalyzed reduction of **9a**

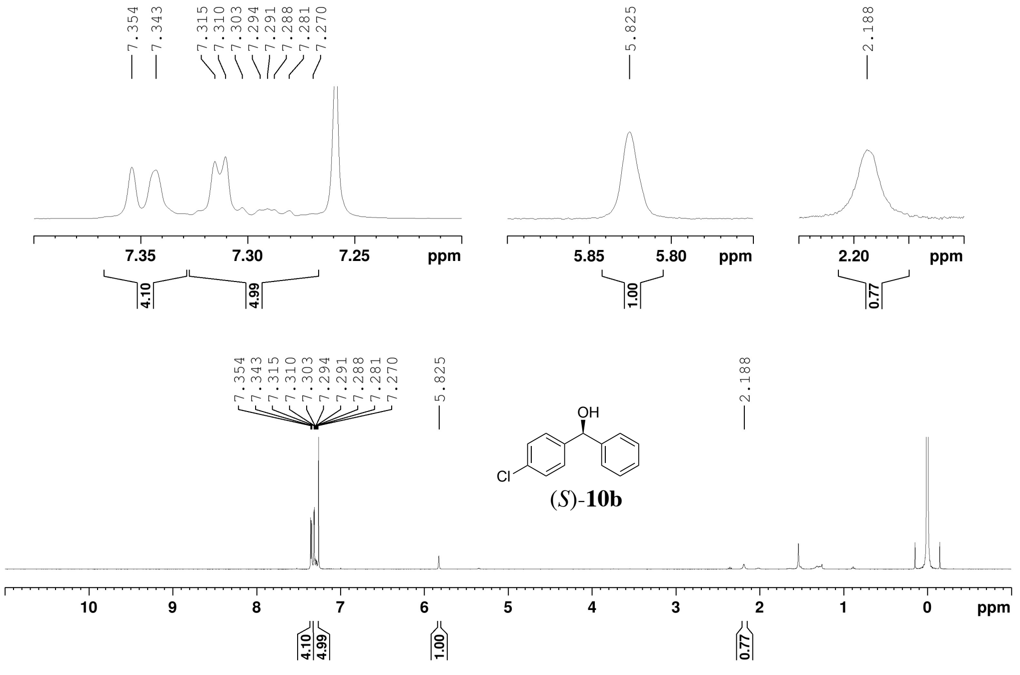

^{1}H-NMR spectrum of product from *Gc*APRD Phe56Ile/Trp288Ala-catalyzed reduction of **10a**

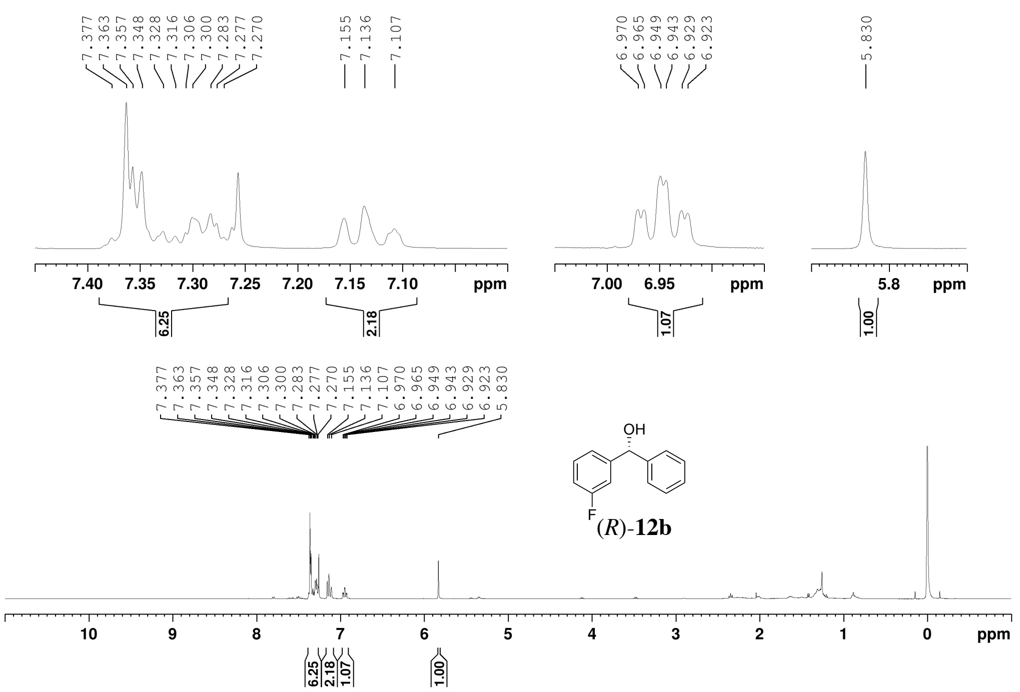

^{1}H-NMR spectrum of product from *Gc*APRD Phe56Ile/Trp288Ala-catalyzed reduction of **12a**

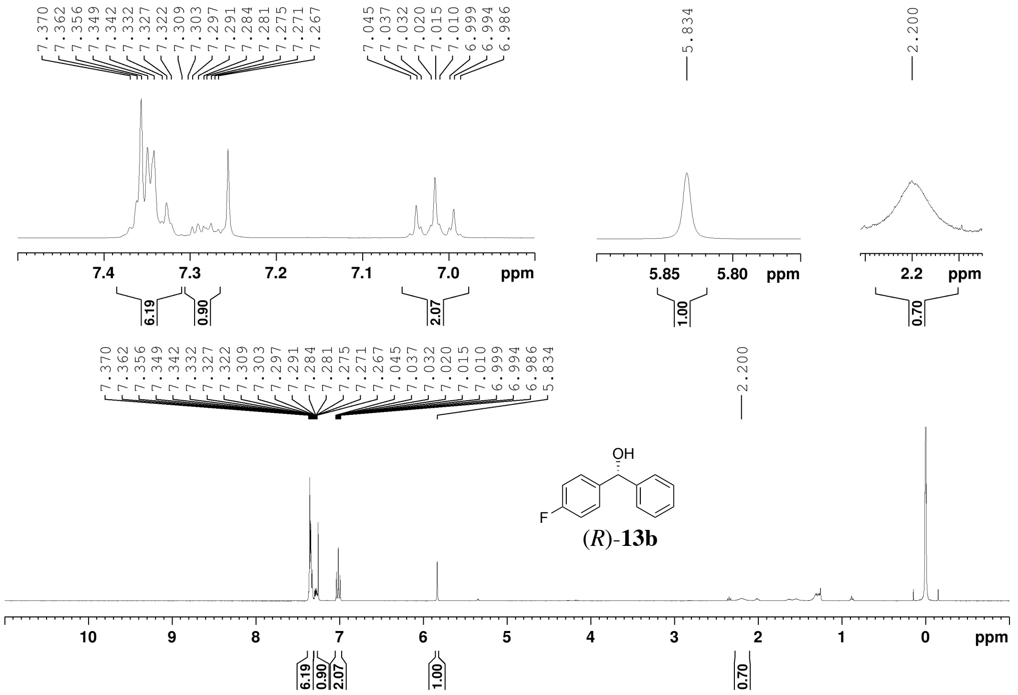

^{1}H-NMR spectrum of product from *Gc*APRD Phe56Ile/Trp288Ala-catalyzed reduction of **13a**

5. ^{1}H-NMR spectra and HPLC chromatograms of products from *Gc*APRD Phe56Ile/Trp288Ala-catalyzed scaled-up reductions

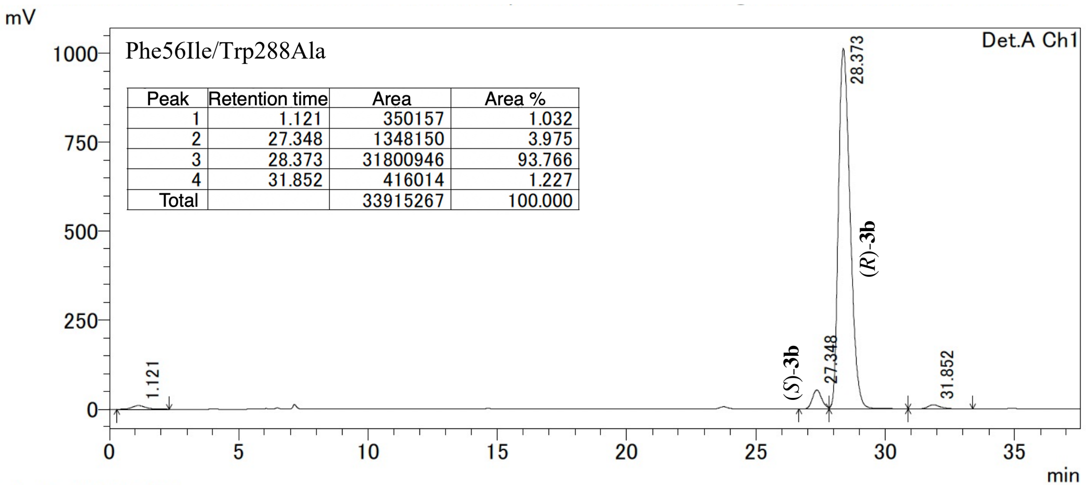

HPLC chromatogram of product from *Gc*APRD Phe56Ile/Trp288Ala-catalyzed scaled-up reduction of **3a**

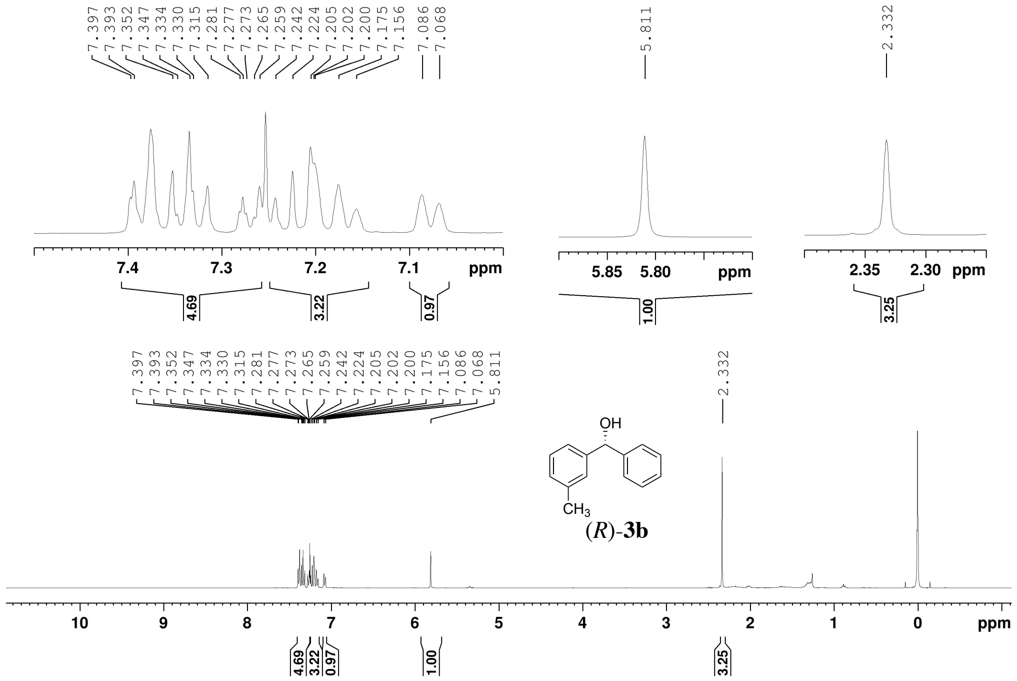

^{1}H-NMR spectrum of product from *Gc*APRD Phe56Ile/Trp288Ala-catalyzed scaled-up reduction of **3a**

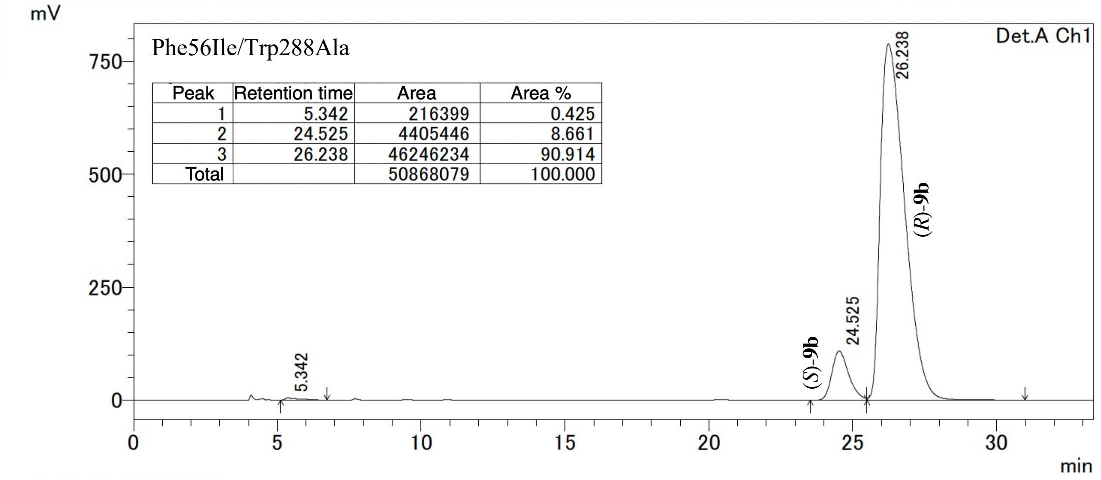

HPLC chromatogram of product from *Gc*APRD Phe56Ile/Trp288Ala-catalyzed scaled-up reduction of **9a**

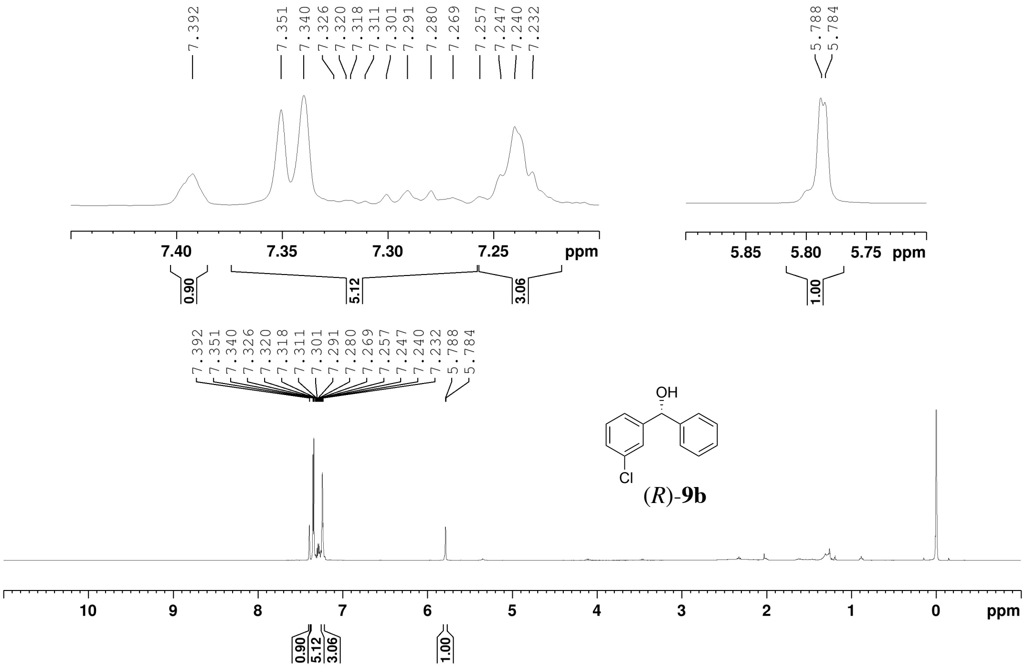

^{1}H-NMR spectrum of product from *Gc*APRD Phe56Ile/Trp288Ala-catalyzed scaled-up reduction of **9a**
