# Supplementary material for: Highly enantioselective reduction of benzophenones by engineered Geotrichum candidum alcohol dehydrogenase
Source: Appl Microbiol Biotechnol. 2026 Jan 29;110(1):48. doi: 10.1007/s00253-026-13717-0 (PMC12858457; doi:10.1007/s00253-026-13717-0)
Supplement: Supplementary file 2 — (PDF 3.23 MB) [file 253_2026_13717_MOESM2_ESM.pdf]

Journal name: Applied Microbiology and Biotechnology

Supplementary Materials

Highly Enantioselective Reduction of Benzophenones by Engineered *Geotrichum candidum* Alcohol Dehydrogenase

Zhongyao Tang[1], Guillermo Germán Otárola Tejada[1,2], Afifa Ayu Koesoema[1], Tomoko Matsuda[1*]

1. Department of Life Science and Technology, School of Life Science and Technology, Institute of Science Tokyo, 4259 Nagatsuta-cho Midori-ku, Yokohama 226-8501, Japan
2. Department of Organic and Inorganic Chemistry, University of Alcalá, Ctra. Madrid-Barcelona km 33.100, Alcalá de Henares, Madrid 28805, Spain

Corresponding author:
Tomoko Matsuda (tmatsuda@bio.titech.ac.jp, +81-45-924-5757)

Table of contents

1. Synthesis of standard *rac*-**3b**, *rac*-**6b**, *rac*-**12b**, and *rac*-**13b** by sodium borohydride reduction 1

2. Small-scale reductions of **1a**-**13a** by *Gc*APRD Trp288Ala and Phe56Ile/Trp288Ala 1

3. Scaled-up reductions of **3a** and **9a** by *Gc*APRD Phe56Ile/Trp288Ala 3

Table S1 The chiral HPLC analysis method and retention time of racemic alcohols 4

Table S2 Summary of yields and enantioselectivities for the small-scale reductions of **1a**-**13a** catalyzed by *Gc*APRD mutants 5

Fig. S1 Restraint parameters used to determine productive poses in docking simulation. 6

Fig. S2 Visualization of steric hindrance to **1a** caused by Trp288 in *Gc*APRD wild type 6

Fig. S3 Comparison of pro-*S* and pro-*R* poses of 3-substituted ligands in the docking model of *Gc*APRD Trp288Ala 7

Fig. S4 Comparison of pro-*S* and pro-*R* poses of 4-substituted ligands in the docking model of *Gc*APRD Trp288Ala 8

Fig. S5 Comparison of pro-*S* and pro-*R* poses of 3-substituted ligands in the docking model of *Gc*APRD Phe56Ile/Trp288Ala 9

Fig. S6 Comparison of pro-*S* and pro-*R* poses of 4-substituted ligands in the docking model of *Gc*APRD Phe56Ile/Trp288Ala 10

Table S3 Docking simulation results of *Gc*APRD Trp288Ala 11

Table S4 Docking simulation results of *Gc*APRD Phe56Ile/Trp288Ala 12

Table S5 Examples of biocatalytic reduction of benzophenone analogs used in this study 13

References 14

1. Synthesis of standard *rac*-3b, *rac*-6b, *rac*-12b, and *rac*-13b by sodium borohydride reduction

$NaBH_4$ (129.4 mg, 3.42 mmol) was added to **3a** (442.8 mg, 2.26 mmol) in dry ethanol (20 mL) and stirred in an ice bath. The suspension was further stirred at room temperature until the reaction was detected to be completed by monitoring with TLC. Then, the reaction was quenched by the addition of 1 N HCl until the pH turned to 7-8. After removing ethanol, the residue was extracted with Et_2O, then washed with saturated $NaHCO_3$. The combined Et_2O layers were dried by $MgSO_4$, and the solvent was evaporated under reduced pressure. The product was purified by silica gel column chromatography (hexane: ethyl acetate, 5:1) to give *rac*-**3b**. *Rac*-**6b**, *rac*-**12b**, and *rac*-**13b** were synthesized with the above procedure. The ^{1}H-NMR spectrum of the products was obtained using 400 MHz Bruker Biospin Avance III 400A spectrometer (Bruker, USA), and compared with the spectra data reported in the literature (Karthikeyan et al. 2010; Brodmann et al. 2012; Gaykar et al. 2018). The results were as follows.

rac-**3b** (406.8 mg, yield 91%, white solid), ^{1}H-NMR (400 MHz, $CDCl_3$): δ=7.40-7.26 (m, 5H), 7.24-7.16 (m, 3H), 7.08 (d, *J*=7.6 Hz, 1H), 5.81 (d, *J*=3.2 Hz, 1H), 2.33 (s, 3H), 2.18 (d, *J*=3.2 Hz, 1H).

rac-**6b** (122.0 mg, yield 93%, white solid), ^{1}H-NMR (400 MHz, $CDCl_3$): δ=7.57 (s, 1H), 7.40-7.27 (m, 7H), 7.20 (t, *J*=7.8 Hz, 1H), 5.80 (d, *J*=2.8 Hz, 1H), 2.22 (d, *J*=3.2 Hz, 1H).

rac-**12b** (96.0 mg, yield 94%, colorless oil), ^{1}H-NMR (400 MHz, $CDCl_3$): δ=7.36-7.26 (m, 6H), 7.16-7.11 (m, 2H), 7.00-6.94 (m, 1H), 5.83 (d, *J*=2.4 Hz, 1H), 2.23 (d, *J*=3.2 Hz, 1H).

rac-**13b** (96.6 mg, yield 90%, colorless oil), ^{1}H-NMR (400 MHz, $CDCl_3$): δ=7.36-7.27 (m, 7H), 7.04-6.99 (m, 2H), 5.83 (s, 1H), 2.20 (d, *J*=2.8 Hz, 1H).

2. Small-scale reductions of 1a-13a by *Gc*APRD Trp288Ala and Phe56Ile/Trp288Ala

Reduction of **1a**

Trp288Ala: **1a** (0.030 mmol) was converted to **1b** (4.3 mg, 0.023 mmol, isolated yield 77%).

Phe56Ile/Trp288Ala: **1a** (0.030 mmol) was converted to **1b** (4.1 mg, 0.022 mmol, isolated yield 74%).

^{1}H-NMR (400 MHz, $CDCl_3$): δ=7.40-7.32 (m, 8H), 7.29-7.27 (m, 2H), 5.86 (s, 1H).

Reduction of **2a**

Trp288Ala: **2a** (0.030 mmol) was not converted to **2b** as far as detected with ^{1}H-NMR (^{1}H-NMR yield < 0.1%).

Phe56Ile/Trp288Ala: **2a** (0.030 mmol) was not converted to **2b** as far as detected with ^{1}H-NMR (^{1}H-NMR yield < 0.1%).

Reduction of **3a**

Trp288Ala: **3a** (0.030 mmol) was converted to (*R*)-**3b** (4.3 mg, 0.022 mmol, isolated yield 72%,

ee 65% (*R*)).

Phe56Ile/Trp288Ala: **3a** (0.030 mmol) was converted to (*R*)-**3b** (5.2 mg, 0.026 mmol, isolated yield 87%, *ee* 89% (*R*)).

^{1}H-NMR (400 MHz, $CDCl_3$): δ=7.40-7.28 (m, 5H), 7.25-7.16 (m, 3H), 7.08 (d, *J*=7.2 Hz, 1H), 5.82 (s, 1H), 2.34 (s, 3H).

Reduction of **4a**

Trp288Ala: **4a** (0.030 mmol) was converted to (*S*)-**4b** (^{1}H-NMR yield 25%, *ee* 18% (*S*)). The product was not isolated.

Phe56Ile/Trp288Ala: **4a** (0.030 mmol) was converted to (*S*)-**4b** (3.6 mg, 0.018 mmol, isolated yield 60%, *ee* 88% (*S*)).

^{1}H-NMR (400 MHz, $CDCl_3$): δ=7.39-7.27 (m, 6H), 7.14 (d, *J*=8.0 Hz, 1H), 5.82 (s, 1H), 2.33 (3, 3H).

Reduction of **5a**

Trp288Ala: **5a** (0.030 mmol) was not converted to **5b** (^{1}H-NMR yield 4%). The product was not isolated.

Phe56Ile/Trp288Ala: **5a** (0.030 mmol) was not converted to **5b** as far as detected with ^{1}H-NMR (^{1}H-NMR yield < 0.1%).

Reduction of **6a**

Trp288Ala: **6a** (0.030 mmol) was converted to (*R*)-**6b** (5.2 mg, 0.020 mmol, isolated yield 66%, *ee* 80% (*R*)).

Phe56Ile/Trp288Ala: **6a** (0.030 mmol) was converted to (*R*)-**6b** (5.7 mg, 0.022 mmol, isolated yield 73%, *ee* 87% (*R*)).

^{1}H-NMR (400 MHz, $CDCl_3$): δ=7.60 (s, 1H), 7.40-7.33 (m, 5H), 7.32-7.28 (m, 2H), 7.20 (t, *J*=7.8 Hz, 1H), 5.81 (s, 1H), 2.21 (brs, 1H).

Reduction of **7a**

Trp288Ala: **7a** (0.030 mmol) was converted to (*S*)-**7b** (^{1}H-NMR yield 5%, *ee* 6% (*S*)). The product was not isolated.

Phe56Ile/Trp288Ala: **7a** (0.030 mmol) was converted to (*S*)-**7b** (4.6 mg, 0.017 mmol, isolated yield 59%, *ee* 92% (*S*)).

^{1}H-NMR (400 MHz, $CDCl_3$): δ=7.46 (dd, *J*=6.6 Hz, 1.8Hz, 2H), 7.35-7.28 (m, 7H), 5.81 (s, 1H), 2.19 (s, 1H).

Reduction of **8a**

Trp288Ala: **8a** (0.030 mmol) was not converted to **8b** as far as detected with ^{1}H-NMR (^{1}H-NMR yield < 0.1%).

Phe56Ile/Trp288Ala: **8a** (0.030 mmol) was not converted to **8b** as far as detected with ^{1}H-NMR (^{1}H-NMR yield < 0.1%).

Reduction of **9a**

Trp288Ala: **9a** (0.030 mmol) was converted to (*R*)-**9b** (3.3 mg, 0.015 mmol, isolated yield 50%, *ee* 74% (*R*)).

Phe56Ile/Trp288Ala: **9a** (0.030 mmol) was converted to (*R*)-**9b** (4.9 mg, 0.022 mmol, isolated yield 75%, *ee* 85% (*R*)).

^{1}H-NMR (400 MHz, $CDCl_3$): *δ*=7.40 (s, 1H), 7.37-7.27 (m, 5H), 7.25-7.24 (m, 3H), 5.81 (s, 1H).

Reduction of **10a**

Trp288Ala: **10a** (0.030 mmol) was converted to (*R*)-**10b** (^{1}H-NMR yield 17%, *ee* 57% (*R*)). The product was not isolated.

Phe56Ile/Trp288Ala: **10a** (0.030 mmol) was converted to (*S*)-**10b** (2.8 mg, 0.013 mmol, isolated yield 43%, *ee* 81% (*S*)).

^{1}H-NMR (400 MHz, $CDCl_3$): *δ*=7.35-7.34 (m, 4H), 7.32-7.27 (m, 5H), 5.83 (s, 1H), 2.19 (s, 1H).

Reduction of **11a**

Trp288Ala: **11a** (0.030 mmol) was not converted to **11b** as far as detected with ^{1}H-NMR (^{1}H-NMR yield < 0.1%).

Phe56Ile/Trp288Ala: **11a** (0.030 mmol) was not converted to **11b** as far as detected with ^{1}H-NMR (^{1}H-NMR yield < 0.1%).

Reduction of **12a**

Trp288Ala: **12a** (0.030 mmol) was converted to (*R*)-**12b** (2.7 mg, 0.013 mmol, isolated yield 45%, *ee* 43% (*R*)).

Phe56Ile/Trp288Ala: **12a** (0.030 mmol) was converted to (*R*)-**12b** (4.4 mg, 0.022 mmol, isolated yield 73%, *ee* 32% (*R*)).

^{1}H-NMR (400 MHz, $CDCl_3$): *δ*=7.38-7.27 (m, 6H), 7.16-7.11 (m, 2H), 6.95 (td, *J*=8.3 Hz, 2.3 Hz, 1H), 5.83 (s, 1H).

Reduction of **13a**

Trp288Ala: **13a** (0.030 mmol) was converted to (*R*)-**13b** (3.2 mg, 0.016 mmol, isolated yield 53%, *ee* 97% (*R*)).

Phe56Ile/Trp288Ala: **13a** (0.030 mmol) was converted to (*R*)-**13b** (4.1 mg, 0.020 mmol, isolated yield 68%, *ee* 55% (*R*)).

^{1}H-NMR (400 MHz, $CDCl_3$): *δ*=7.37-7.32 (m, 6H), 7.31-7.27 (m, 1H), 7.05-6.99 (m, 2H), 5.83 (s, 1H), 2.20 (brs, 1H).

3. Scaled-up reductions of 3a and 9a by *Gc*APRD Phe56Ile/Trp288Ala

3a (65.9 mg, 0.34 mmol) was converted to (*R*)-**3b** (63.6 mg, 0.32 mmol, yield 96%, white solid), $[\alpha]_D^{26}$= 10.9 (c=1.0, $CHCl_3$, 92% *ee*) (*lit.* (Yao et al. 2021) $[\alpha]_D^{20}$ = 4.8 (c = 0.4, $CHCl_3$, 88% *ee* (*R*)); ^{1}H-NMR (400 MHz, $CDCl_3$): *δ*=7.40-7.26 (m, 5H), 7.24-7.16 (m, 3H), 7.08 (d, *J*=7.2 Hz,

1H), 5.81 (s, 1H), 2.33 (s, 1H).

9a (74.7 mg, 0.34 mmol) was converted to (*R*)-**9b** (62.4 mg, 0.29 mmol, yield 83%, colorless oil), $[\alpha]_D^{27}$= -61.9 (c=1.0, $CHCl_3$, 83% *ee*) (*lit.* (Tsuda et al. 2022) $[\alpha]_D^{23}$ = –34.0 (c = 0.4, $CHCl_3$, 94% *ee* (*R*)); ^{1}H-NMR (400 MHz, $CDCl_3$): δ=7.39 (s, 1H), 7.35-7.27 (m, 5H), 7.26-7.23 (m, 3H), 5.79 (brd, J=1.6 Hz, 1H).

Table S1 The chiral HPLC analysis method and retention time of racemic alcohols

Compound	Conditions	Retention time (min)[a]		Reference
		S	*R*	
rac-**3b**	A	27.3	28.3	(Umeda and Studer 2008)
rac-**4b**	B	16.4	15.7	
rac-**6b**	C	23.3	27.2	(Lu et al. 2019)
rac-**7b**	B	17.9	16.7	(Karthikeyan et al. 2010)
rac-**9b**	C	24.9	27.3	(Wu et al. 2005)
rac-**10b**	B	17.0	16.0	(Yang et al. 2009)
rac-**12b**	D	18.8	20.5	(Zheng et al. 2013)
rac-**13b**	E	17.7	21.7	(Yang et al. 2014)

HPLC conditions:

A: CHIRALPAK IA-3 (4.6 mm × 250 mm × 3 μm, Daicel, Japan), hexane: 2-propanol=97:3, 0.5 mL/min, 230 nm, room temperature.

B: CHIRALPAK IA-3 (4.6 mm × 250 mm × 3 μm, Daicel, Japan), hexane: 2-propanol=90:10, 0.5 mL/min, 230 nm, room temperature.

C: CHIRALCEL OD-H (4.6 mm × 250 mm × 5 μm, Daicel, Japan), hexane: 2-propanol=95:5, 0.8 mL/min, 230 nm, room temperature.

D: CHIRALCEL OB-H (4.6 mm × 250 mm × 5 μm, Daicel, Japan), hexane: 2-propanol=90:10, 0.8 mL/min, 230 nm, room temperature.

E: CHIRALCEL OB-H (4.6 mm × 250 mm × 5 μm, Daicel, Japan), hexane: 2-propanol=80:20, 0.8 mL/min, 230 nm, room temperature.

[a]The absolute configuration of *R* and *S* enantiomers was determined by referring to the literature.

Table S2 Summary of yields and enantioselectivities for the small-scale reductions of **1a**-**13a** catalyzed by *Gc*APRD mutants shown in Fig.2.

Substrate	Trp288Ala		Phe56Ile/Trp288Ala	
	Yield[a] (%)	*ee*[b] (%)	Yield[a] (%)	*ee*[b] (%)
**1a**	80	NA	83	NA
**2a**	ND	ND	ND	ND
**3a**	85	65 (*R*)	93	89 (*R*)
**4a**	25	18 (*S*)	65	88 (*S*)
**5a**	4	ND	ND	ND
**6a**	79	80 (*R*)	83	87 (*R*)
**7a**	5	6 (*S*)	69	92 (*S*)
**8a**	ND	ND	ND	ND
**9a**	59	74 (*R*)	81	85 (*R*)
**10a**	17	57 (*R*)	58	81 (*S*)
**11a**	ND	ND	ND	ND
**12a**	61	43 (*R*)	76	32 (*R*)
**13a**	54	97 (*R*)	68	55 (*R*)

Small-scale reductions were performed in HEPES-NaOH buffer (100 mM, pH 7.2, 12.0 mL) consisting of 2-propanol (15% v/v), substrate (2.5 mM), and whole cells (2.0 g wet weight) at 30 °C with a shaking speed of 250 rpm for 24 h.

[a]The yield was determined by ^{1}H-NMR analysis. The signal of the product used for the calculation was at 5.8 ppm (1H, singlet). The signal of the internal standard, 1,4-dioxane, used for the calculation was at 3.7 ppm (8H, singlet).

[b]The enantiomeric excess (*ee*) was determined by chiral HPLC analysis.

NA: Not applicable

ND: Not determined due to the low yield

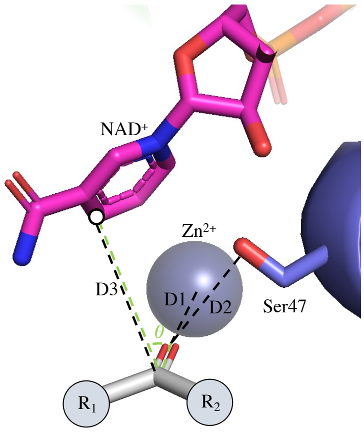

- The ideal distance of ligand carbonyl oxygen to Zn^{2+} (D1): < 2.5 Å
- The ideal distance of ligand carbonyl oxygen to hydroxy oxygen of Ser47 (D2): <3.0 Å
- The ideal distance of ligand carbonyl carbon to C4 of NAD(H) (D3): < 4.0 Å
- The ideal angle of C4 of NAD(H)-ligand carbonyl carbon-ligand carbonyl oxygen (θ): 70°~90°

Fig. S1 Restraint parameters used to determine productive poses in docking simulation (light gray stick: ligand; pink: NAD(H); purple: Ser47; white circle: C4 of NAD(H); gray: catalytic zinc) (Koesoema et al. 2019).

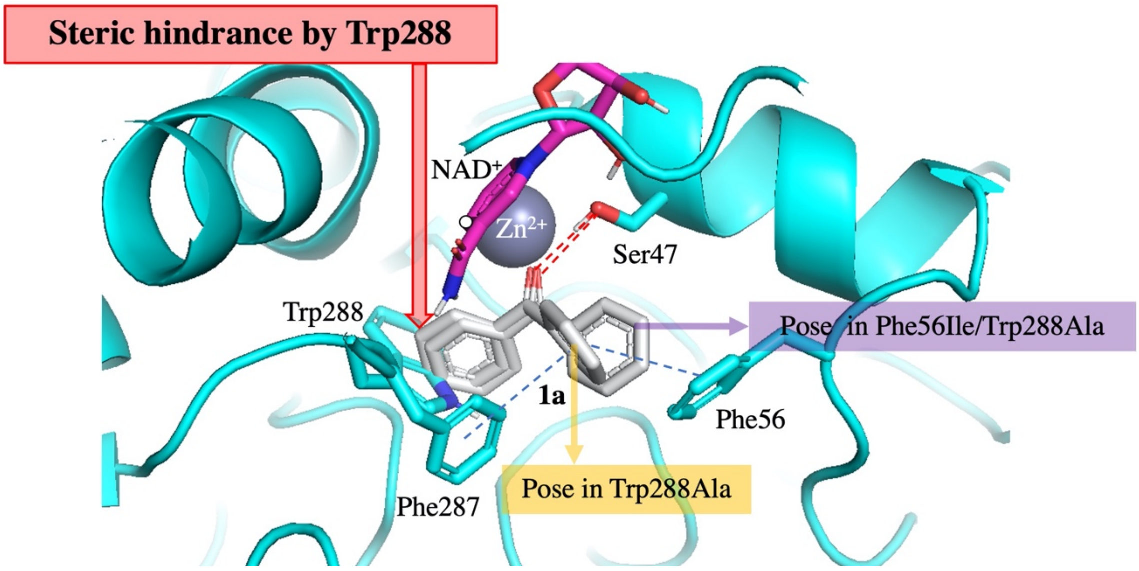

Fig. S2 Visualization of steric hindrance to **1a** caused by Trp288 in *Gc*APRD wild type. **1a** poses obtained in the docking simulation using Trp288Ala and Phe56Ile/Trp288Ala were superimposed onto the structure of the wild type (light gray stick: ligand; pink stick: NAD(H); white circle: C4 of NADH; gray sphere: catalytic zinc; blue dashed line: π-π stacking; red dashed line: hydrogen bond).

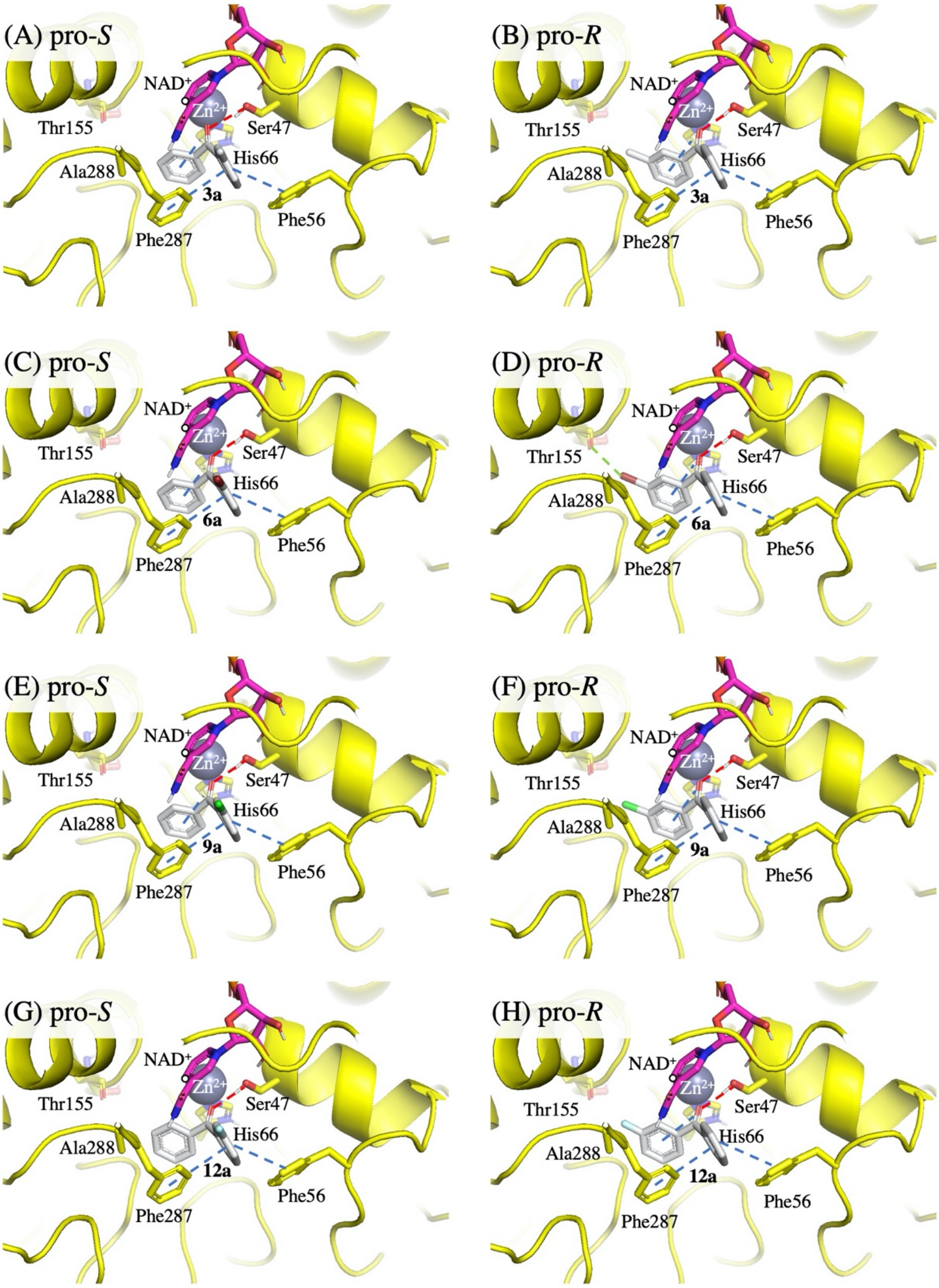

Fig. S3 Comparison of pro-*S* and pro-*R* poses of 3-substituted ligands in the docking model of *Gc*APRD Trp288Ala: (A) pro-*S* pose of **3a**; (B) pro-*R* pose of **3a**; (C) pro-*S* pose of **6a**; (D) pro-*R* pose of **6a**; (E) pro-*S* pose of **9a**; (F) pro-*R* pose of **9a**; (G) pro-*S* pose of **12a**; (H) pro-*R* pose of **12a** (light gray stick: ligand; pink stick: NAD(H); white circle: C4 of NADH; gray sphere: catalytic zinc; blue dashed line: π-π stacking; red dashed line: hydrogen bond; green dashed line: halogen bond).

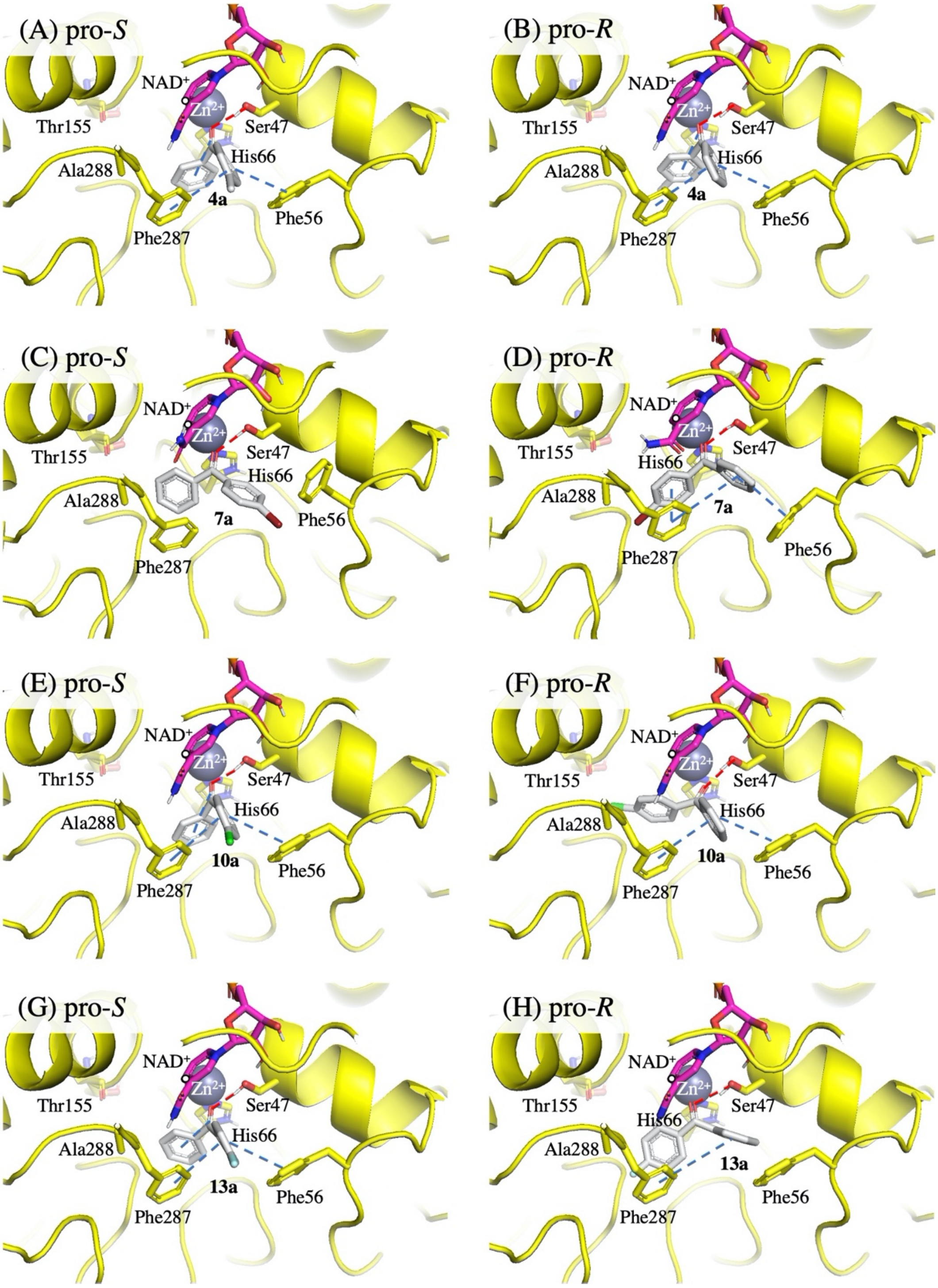

Fig. S4 Comparison of pro-*S* and pro-*R* poses of 4-substituted ligands in the docking model of *Gc*APRD Trp288Ala: (A) pro-*S* pose of **4a**; (B) pro-*R* pose of **4a**; (C) pro-*S* pose of **7a**; (D) pro-*R* pose of **7a**; (E) pro-*S* pose of **10a**; (F) pro-*R* pose of **10a**; (G) pro-*S* pose of **13a**; (H) pro-*R* pose of **13a** (light gray stick: ligand; pink stick: NAD(H); white circle: C4 of NADH; gray sphere: catalytic zinc; blue dashed line: π-π stacking; red dashed line: hydrogen bond; green dashed line: halogen bond; (G) and (H): taken from Fig. 4).

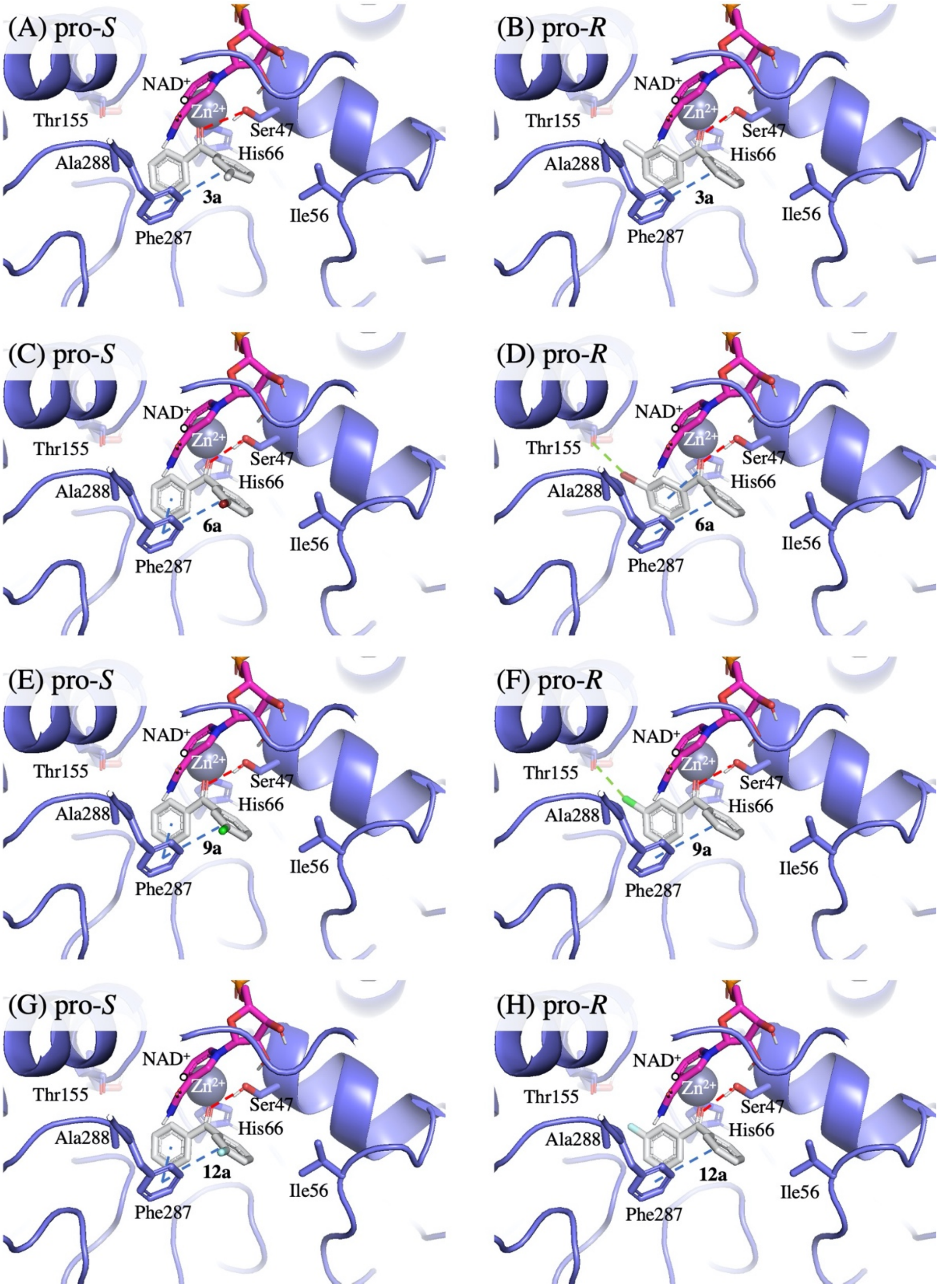

Fig. S5 Comparison of pro-*S* and pro-*R* poses of 3-substituted ligands in the docking model of *Gc*APRD Phe56Ile/Trp288Ala: (A) pro-*S* pose of **3a**; (B) pro-*R* pose of **3a**; (C) pro-*S* pose of **6a**; (D) pro-*R* pose of **6a**; (E) pro-*S* pose of **9a**; (F) pro-*R* pose of **9a**; (G) pro-*S* pose of **12a**; (H) pro-*R* pose of **12a** (light gray stick: ligand; pink stick: NAD(H); white circle: C4 of NADH; gray sphere: catalytic zinc; blue dashed line: π-π stacking; red dashed line: hydrogen bond; green dashed line: halogen bond; (E) and (F):taken from Fig. 5).

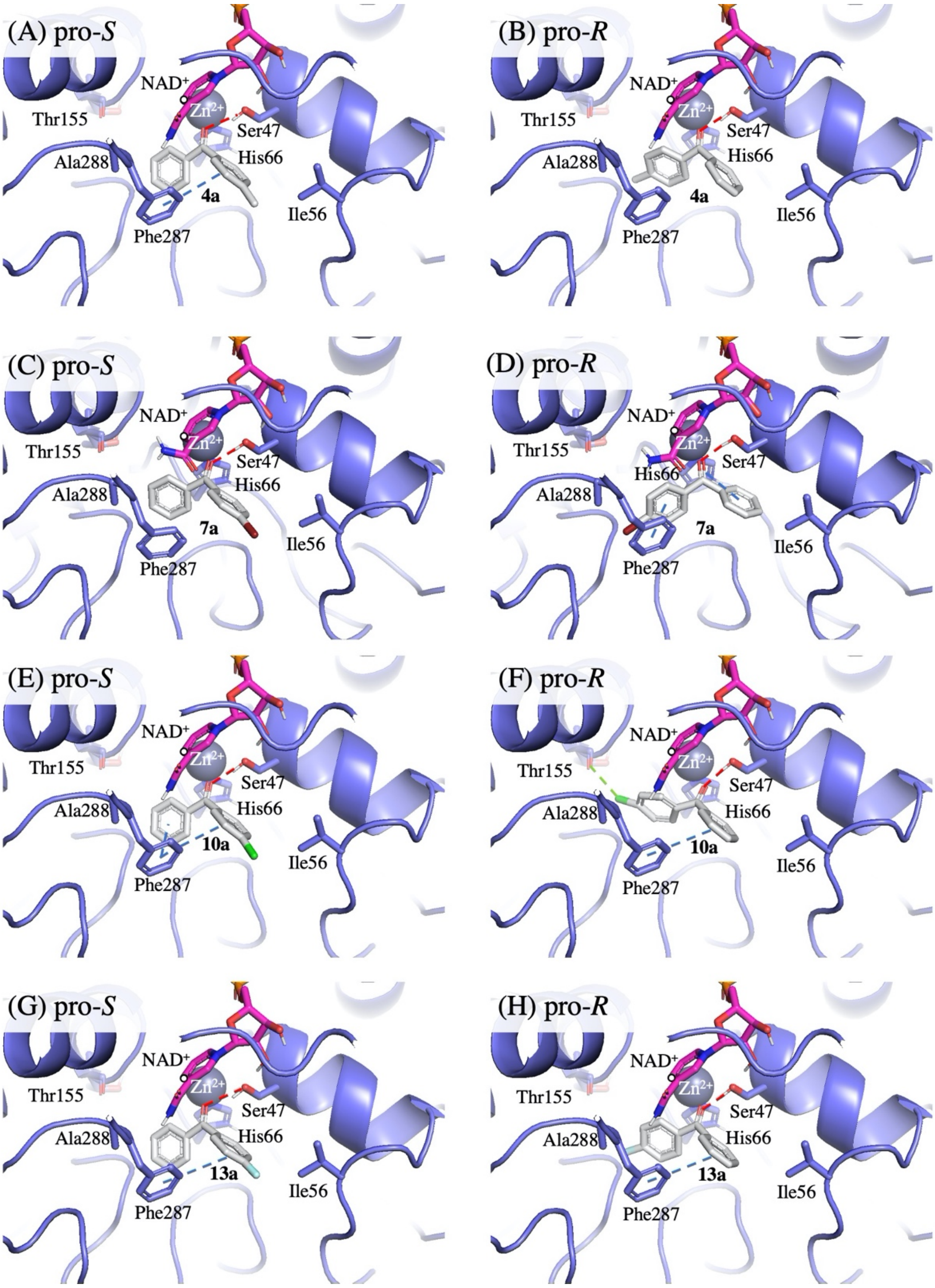

Fig. S6 Comparison of pro-*S* and pro-*R* poses of 4-substituted ligands in the docking model of *Gc*APRD Phe56Ile/Trp288Ala: (A) pro-*S* pose of **4a**; (B) pro-*R* pose of **4a**; (C) pro-*S* pose of **7a**; (D) pro-*R* pose of **7a**; (E) pro-*S* pose of **10a**; (F) pro-*R* pose of **10a**; (G) pro-*S* pose of **13a**; (H) pro-*R* pose of **13a** (light gray stick: ligand; pink stick: NAD(H); white circle: C4 of NADH; gray sphere: catalytic zinc; blue dashed line: π-π stacking; red dashed line: hydrogen bond; green dashed line: halogen bond; (E) and (F): taken from Fig. 5).

Table S3 Docking simulation results[a] of *Gc*APRD Trp288Ala

Ligand	Binding pose	Yield of enantiomer (%)	Binding score (kcal/mol)	D1 (Å)	D2 (Å)	D3 (Å)	θ (°)	Strain energy (kcal/mol)
**1a**	NA	80	-7.2	2.1	2.8	3.9	56.7	4.7
**3a**	pro-*S*	15	-7.5	2.1	2.9	3.7	39.1	3.9
	pro-*R*	70	**-7.7**	2.1	2.8	4.0	52.2	4.2
**4a**	pro-*S*	15	**-7.1**	2.1	2.8	4.0	51.3	3.2
	pro-*R*	10	-6.7	2.1	2.5	3.8	42.8	3.8
**6a**	pro-*S*	8.0	-7.4	2.1	2.8	3.9	54.6	4.0
	pro-*R*	71	**-7.7**	2.1	2.8	4.0	52.7	4.3
**7a**[b]	pro-*S*	2.6	**-8.6**	2.0	3.0	3.9	57.3	NA
	pro-*R*	2.3	-8.1	2.2	2.9	3.7	54.1	NA
**9a**	pro-*S*	7.7	-7.5	2.1	2.8	3.9	54.7	3.9
	pro-*R*	52	**-7.8**	2.1	2.8	4.0	52.4	4.2
**10a**	pro-*S*	3.7	-7.0	2.1	2.8	4.0	52.8	2.4
	pro-*R*	14	-6.2	2.1	2.8	4.2	**80.5**	5.2
**12a**	pro-*S*	17	-7.1	2.1	2.8	3.9	56.1	4.6
	pro-*R*	44	**-7.3**	2.0	2.9	3.9	57.6	4.6
**13a**	pro-*S*	0.8	-7.3	2.1	2.8	4.0	50.5	3.9
	pro-*R*	54	-7.1	2.1	2.9	3.7	35.3	**0.9**

[a]The best binding poses were highlighted in blue (pro-*S*) and orange (pro-*R*), and the key parameters used to determine the favorable poses were in bold. The color intensity reflects the enantioselectivity determined by the experiments.

[b]For **7a**, the binding poses were obtained using induced fit docking (IFD), and the strain energy could not be computed using the same procedure as standard docking simulation.

NA: Not applicable

Table S4 Docking simulation results[a] of *Gc*APRD Phe56Ile/Trp288Ala

Ligand	Binding pose	Yield of enantiomer (%)	Binding score (kcal/mol)	D1 (Å)	D2 (Å)	D3 (Å)	*θ* (°)	Strain energy (kcal/mol)
**1a**	NA	83	-7.2	2.1	2.8	4.1	47.9	0.4
**3a**	pro-*S*	5.2	-8.2	2.0	3.3	3.7	41.8	1.3
	pro-*R*	88	**-8.6**	2.1	**2.8**	3.9	55.3	2.1
**4a**	pro-*S*	61	**-8.4**	2.1	2.9	3.7	44.7	**1.6**
	pro-*R*	4.0	-7.3	2.3	2.5	3.9	50.8	3.9
**6a**	pro-*S*	5.3	-8.4	2.1	2.8	3.9	57.0	1.4
	pro-*R*	78	**-8.8**	2.1	2.8	4.1	49.3	2.8
**7a**[b]	pro-*S*	67	**-8.3**	2.0	2.9	4.0	66.7	NA
	pro-*R*	2.8	-8.1	2.2	3.0	4.1	30.4	NA
**9a**	pro-*S*	6.1	-8.4	2.1	2.9	3.7	45.2	1.5
	pro-*R*	75	**-8.7**	2.1	2.9	3.7	43.4	1.5
**10a**	pro-*S*	53	**-8.4**	2.1	2.8	3.9	56.4	**1.5**
	pro-*R*	5.6	-7.2	2.3	2.7	4.1	73.3	6.3
**12a**	pro-*S*	26	-8.3	2.1	2.8	3.9	56.1	1.3
	pro-*R*	50	-8.3	2.1	2.8	3.9	54.8	1.4
**13a**	pro-*S*	16	-8.2	2.1	2.9	3.7	45.0	1.4
	pro-*R*	53	-7.8	2.2	**2.5**	3.9	57.0	2.7

[a]The best binding poses were highlighted in blue (pro-*S*) and orange (pro-*R*), and the key parameters used to determine the favorable poses were in bold. The color intensity reflects the enantioselectivity determined by the experiments.

[b]For **7a**, the binding poses were obtained using induced fit docking (IFD), and the strain energy could not be computed using the same procedure as standard docking simulation.

NA: Not applicable

Table S5 Examples of biocatalytic reduction of benzophenone analogs used in this study

Type	Enzyme (Organism)	Substrate	Yield/Conversion[a] (%)	Configuration	*ee*[b] (%)	Reference
MDR	*GcAPRD* (*Geotrichum candidum*)	**3a** (3-CH_3)	93	*R*	89	This study
		**4a** (4-CH_3)	65	*S*	88	
		**6a** (3-Br)	83	*R*	87	
		**7a** (4-Br)	69	*S*	92	
		**9a** (3-Cl)	81	*R*	85	
		**10a** (4-Cl)	58	*S*	81	
				R	57	
		**12a** (3-F)	76	*R*	43	
		**13a** (4-F)	68	*R*	97	
	*Tb*SADH (*Thermoanaerobacter brockii*)	**10a** (4-Cl)	41	*R*	> 99	(Liu et al. 2019; Qu et al. 2019)
SDR	*Kp*ADH (*Kluyveromyces polyspora*)	**7a** (4-Br)	NA	*S*	99.9	(Wang et al. 2018; Xu et al. 2018; Zhou et al. 2018; Xu et al. 2020)
				R	80[c]	
		**10a** (4-Cl)	62.2	*S*	97.5	
				R	91.7	
		**13a** (4-F)	99.0	*S*	10[c]	
				R	61.7	
	*Lk*ADH (*Lactobacillus kefiri*)	**3a** (3-CH_3)	NA	*R*	54.0	(Wu et al. 2020; Wu et al. 2021)
		**4a** (4-CH_3)	NA	*S*	51.7	
		**6a** (3-Br)	NA	*S*	95.3	
		**7a** (4-Br)	NA	*R*	90.5	
		**9a** (3-Cl)	NA	*S*	70	
			NA	*R*	68[c]	
		**10a** (4-Cl)	47.9	*R*	> 99.9	
		**13a** (4-F)	NA	*R*	40.3	
	*Km*CR2 (*Kluyveromyces marxianus*)	**3a** (3-CH_3)	> 99	*R*	74	(Li et al. 2019)
		**4a** (4-CH_3)	> 99	*S*	97	
		**7a** (4-Br)	> 99	*S*	99	
		**9a** (3-Cl)	> 99	*R*	87	
		**10a** (4-Cl)	> 99	*S*	98	
		**12a** (3-F)	> 99	*R*	NA	
		**13a** (4-F)	> 99	*S*	81	

[a]The highest yields (this study) or conversions (literature) were listed.

[b]The highest *ee* values for the corresponding enantiomers were listed.

[c]Approximate values estimated from the figures.

NA: Not available in the corresponding references.

References

Brodmann T, Koos P, Metzger A, Knochel P, Ley S V. (2012) Continuous preparation of arylmagnesium reagents in flow with inline IR monitoring. Org Process Res Dev 16:1102–1113. https://doi.org/10.1021/op200275d

Gaykar RN, Bhunia A, Biju AT (2018) Employing arynes for the generation of aryl anion equivalents and subsequent reaction with aldehydes. J Org Chem 83:11333–11340. https://doi.org/10.1021/acs.joc.8b01549

Karthikeyan J, Jeganmohan M, Cheng C (2010) Cobalt-catalyzed addition reaction of organoboronic acids with aldehydes: highly enantioselective synthesis of diarylmethanols. Chem - A Eur J 16:8989–8992. https://doi.org/10.1002/chem.201001160

Koesoema AA, Sugiyama Y, Xu Z, Standley DM, Senda M, Senda T, Matsuda T (2019) Structural basis for a highly (*S*)-enantioselective reductase towards aliphatic ketones with only one carbon difference between side chain. Appl Microbiol Biotechnol 103:9543–9553. https://doi.org/10.1007/s00253-019-10093-w

Li Z, Wang Z, Wang Y, Wu X, Lu H, Huang Z, Chen F (2019) Substituent position-controlled stereoselectivity in enzymatic reduction of diaryl- and aryl(heteroaryl)methanones. Adv Synth Catal 361:1859–1865. https://doi.org/10.1002/adsc.201801543

Liu B, Qu G, Li JK, Fan W, Ma JA, Xu Y, Nie Y, Sun Z (2019) Conformational dynamics-guided loop engineering of an alcohol dehydrogenase: capture, turnover and enantioselective transformation of difficult-to-reduce ketones. Adv Synth Catal 361:3182–3190. https://doi.org/10.1002/adsc.201900249

Lu Z, Zhang H, Yang Z, Ding N, Meng L, Wang J (2019) Asymmetric hydrophosphination of heterobicyclic alkenes: facile access to phosphine ligands for asymmetric catalysis. ACS Catal 9:1457–1463. https://doi.org/10.1021/acscatal.8b04787

Qu G, Liu B, Jiang Y, Nie Y, Yu H, Sun Z (2019) Laboratory evolution of an alcohol dehydrogenase towards enantioselective reduction of difficult-to-reduce ketones. Bioresour Bioprocess 6:18. https://doi.org/10.1186/s40643-019-0253-9

Tsuda S, Asahi K, Takahashi R, Yamauchi H, Ueda R, Iwasaki T, Fujiwara SI, Kambe N (2022) Bio-inspired asymmetric aldehyde arylations catalyzed by rhodium-cyclodextrin self-inclusion complexes. Org Biomol Chem 20:801–807. https://doi.org/10.1039/d1ob02014e

Umeda R, Studer A (2008) Ag-catalyzed stereoselective cyclohexadienyl transfer: a novel entry into arylphenylmethanols. Org Lett 10:993–996. https://doi.org/10.1021/ol703080a

Wang Y, Dai W, Liu Y, Zhang Z, Zhou J, Xu G, Ni Y (2018) Fine tuning the enantioselectivity and substrate specificity of alcohol dehydrogenase from *Kluyveromyces polysporus* by single residue at 237. Catal Commun 108:1–6. https://doi.org/10.1016/j.catcom.2018.01.012

Wu K, Yan J, Wang X, Yin X, Shi G, Yang L, Li F, Huang J, Shao L (2021) Efficient synthesis of bepotastine and cloperastine intermediates using engineered alcohol dehydrogenase with

a hydrophobic pocket. Appl Microbiol Biotechnol 105:5873–5882. https://doi.org/10.1007/S00253-021-11413-9

Wu K, Yang Z, Meng X, Chen R, Huang J, Shao L (2020) Engineering an alcohol dehydrogenase with enhanced activity and stereoselectivity toward diaryl ketones: reduction of steric hindrance and change of the stereocontrol element. Catal Sci Technol 10:1650–1660. https://doi.org/10.1039/c9cy02444a

Wu X, Liu X, Zhao G (2005) Catalyzed asymmetric aryl transfer reactions to aldehydes with boroxines as aryl source. Tetrahedron Asymmetry 16:2299–2305. https://doi.org/10.1016/j.tetasy.2005.06.010

Xu G, Dai W, Wang Y, Zhang L, Sun Z, Zhou J, Ni Y (2020) Molecular switch manipulating Prelog priority of an alcohol dehydrogenase toward bulky-bulky ketones. Mol Catal 484:110741. https://doi.org/10.1016/j.mcat.2019.110741

Xu G, Wang Y, Tang M, Zhou J, Zhao J, Han R, Ni Y (2018) Hydroclassified combinatorial saturation mutagenesis: reshaping substrate binding pockets of *Kp*ADH for enantioselective reduction of bulky-bulky ketones. ACS Catal 8:8336–8345. https://doi.org/10.1021/acscatal.8b02286

Yang X, Hirose T, Zhang G (2009) Catalytic enantioselective arylation of aryl aldehydes by chiral aminophenol ligands. Tetrahedron Asymmetry 20:415–419. https://doi.org/10.1016/j.tetasy.2009.01.025

Yang Y, Liu Y, Zhang L, Jia Y, Wang P, Zhuo F, An X (2014) Aryl bromides as inexpensive starting materials in the catalytic enantioselective arylation of aryl aldehydes: the additive TMEDA enhances the enantioselectivity. J Org Chem 79:10696–10702. https://doi.org/dx.doi.org/10.1021/jo502070r

Yao C, Chen Y, Sun R, Wang C, Huang Y, Li L, Li YM (2021) Binaphthyl-prolinol chiral ligands: design and their application in enantioselective arylation of aromatic aldehydes. Org Biomol Chem 19:3644–3655. https://doi.org/10.1039/d1ob00289a

Zheng L, Jiang K, Deng Y, Bai X, Gao G, Gu F, Xu L (2013) Synthesis of Ar-BINMOL ligands by [1,2]-wittig rearrangement to probe their catalytic activity in 1,2-addition reactions of aldehydes with Grignard reagents. European J Org Chem 748–755. https://doi.org/10.1002/ejoc.201201301

Zhou J, Wang Y, Xu G, Wu L, Han R, Schwaneberg U, Rao Y, Zhao Y, Zhou J, Ni Y (2018) Structural insight into enantioselective inversion of an alcohol dehydrogenase reveals a “polar gate” in stereorecognition of diaryl ketones. J Am Chem Soc 140:12645–12654. https://doi.org/10.1021/jacs.8b08640
